# Single‐Molecule Counting of High‐Sensitivity Troponin I in Patients Referred for Diagnostic Angiography: Results From the CASABLANCA (Catheter Sampled Blood Archive in Cardiovascular Diseases) Study

**DOI:** 10.1161/JAHA.117.007975

**Published:** 2018-03-08

**Authors:** Cian P. McCarthy, Nasrien E. Ibrahim, Asya Lyass, Yiwei Li, Hanna K. Gaggin, Mandy L. Simon, Renata Mukai, Parul Gandhi, Noreen Kelly, Shweta R. Motiwala, Roland R. J. van Kimmenade, Joseph M. Massaro, Ralph B. D'Agostino, James L. Januzzi

**Affiliations:** ^1^ Department of Medicine Massachusetts General Hospital Boston MA; ^2^ Division of Cardiology Massachusetts General Hospital Boston MA; ^3^ Cardiometabolic Trials Baim Institute for Clinical Research Boston MA; ^4^ Division of Cardiology VA Connecticut Healthcare System and Yale University New Haven CT; ^5^ Division of Cardiology Brigham and Women's Hospital Boston MA; ^6^ Division of Cardiology Beth Israel Deaconess Medical Center Boston MA; ^7^ Division of Cardiology Maastricht University Medical Centre Maastricht the Netherlands; ^8^ Department of Biostatistics Boston University School of Public Health Boston MA

**Keywords:** biomarkers, coronary artery disease, high‐sensitivity, troponin, Biomarkers, Prognosis, Angiography

## Abstract

**Background:**

The meaning of high‐sensitivity troponin I (hsTnI) concentrations in patients without acute myocardial infarction (MI) requires clarity. We hypothesized that among patients referred for diagnostic coronary angiography without acute MI, hsTnI concentrations would correlate with prevalent coronary artery disease (CAD) and predict incident cardiovascular events and mortality.

**Methods and Results:**

We measured hsTnI using a single‐molecule counting assay (99th percentile, 6 ng/L) in samples from 991 patients obtained at the time of angiography. Concentrations of hsTnI were assessed relative to the severity of CAD and prognosis during mean follow‐up of 3.7 years. Median hsTnI concentration was 4.19 ng/L; 38% of patients had hsTnI concentrations ≥99th percentile. Across increasing hsTnI quartiles, patients had higher prevalence of angiographic CAD; in multivariate models, hsTnI ≥99th percentile independently predicted obstructive CAD (odds ratio: 2.57; *P*<0.001) and incident MI (hazard ratio [HR]: 2.68; *P*<0.001), cardiovascular death (HR: 2.29; *P*=0.001), and all‐cause death (HR: 1.84; *P*=0.004). In those with >70% coronary stenosis, hsTnI ≥99th percentile independently predicted incident MI (HR: 1.87; *P*=0.01), cardiovascular mortality (HR: 2.74; *P*=0.001), and the composite end point of MI and all‐cause death (HR: 2.06; *P*<0.001). In participants with coronary stenosis <70%, hsTnI ≥99th percentile even more strongly predicted incident MI (HR: 8.41; *P*<0.001), cardiovascular mortality (HR: 3.60; *P*=0.03), and the composite end point of MI and all‐cause death (HR: 3.62; *P*<0.001).

**Conclusions:**

In a large prospective cohort of patients who were free of prevalent MI and undergoing diagnostic coronary angiography, hsTnI concentrations were associated with higher prevalence of CAD and predicted incident MI, cardiovascular death, and all‐cause death.

**Clinical Trial Registration:**

URL: http://www.clinicaltrials.gov. Unique identifier: NCT00842868.

**Clinical Perspective**

**What Is New?**

- In patients undergoing diagnostic coronary angiography, high‐sensitivity troponin I concentrations were associated with higher prevalence of coronary artery disease and independently predicted cardiovascular events in patients with obstructive and nonobstructive coronary artery disease.

**What Are the Clinical Implications?**

- These data highlight the diagnostic and prognostic utility of high‐sensitivity troponin I in patients with coronary artery disease for identifying patients who may benefit from more aggressive medical therapy to reduce atherothrombotic risk.

## Introduction

Cardiovascular disease is a major modern health concern; it is projected that by 2030, >23.3 million people globally will die annually from acute myocardial infarction (MI), stroke, and other cardiovascular diseases.^1^ Patients with coronary artery disease (CAD) are naturally at higher risk of developing cardiovascular events compared with the general population; however, even among patients with CAD, incidence of cardiovascular events can vary substantially. In patients with stable CAD enrolled in the REACH (Reduction of Atherothrombosis for Continued Health) registry, for example, the annual mortality rate ranged from 0.63% in patients with nonobstructive CAD to 3.8% in higher risk patients.^2^ Consequently, efforts to improve risk stratification, permitting appropriate intervention for each individual, are merited.

In recent years, biomarkers have been identified as important tools for prognostication in patients with CAD beyond that with conventional risk factors, and most data focused on the value of biomarkers for those with acute syndromes such as MI.^3^, ^4^, ^5^, ^6^ The most widely studied biomarkers for evaluation of acute complications of CAD are cardiac troponins I and T. Recent refinements in assay technology have led to the development of highly sensitive assays for measurement of cardiac troponin. Although they have advantages over conventional troponin methods for earlier and more rapid identification of acute MI, it is of interest that high‐sensitivity troponin (hsTn) assays are also able to detect circulating concentrations of the biomarker in patients not previously thought to have acute myonecrosis; such increased sensitivity leads to reclassification from unstable angina to acute MI in a significant percentage of patients, as well as detection of myocardial injury or necrosis in those not previously considered to have an acute coronary syndrome. Nevertheless, elevations in troponin may not be exclusively explained by myonecrosis.^7^ This detection of hsTn elevation in those without MI has led to some consternation about ambiguity regarding the meaning of such a situation and has led to the increased use of terms such as myocardial “injury” to explain this finding; better definition of the description of patients without MI that have higher hsTn would be of use. Beyond its diagnostic use, hsTn is also recognized as a prognostic biomarker in various cardiac diseases.^8^, ^9^ It remains unclear, however, how this prognostic value varies based on presence and severity of CAD; few studies have examined the prognostic utility of hsTn in patients with varying magnitude of atherosclerotic disease,^10^, ^11^, ^12^ in part because of a lack of well‐defined coronary anatomy in these trials.

Given these open questions, we examined the diagnostic and prognostic utility of hsTnI in 991 patients without acute MI referred for diagnostic coronary angiography for various indications who were enrolled in the CASABLANCA (Catheter Sampled Blood Archive in Cardiovascular Diseases) study (http://Clinical Trials.Gov identifier NCT00842868).^13^ We hypothesized that hsTnI concentrations would correlate with prevalent CAD and predict incident cardiovascular events and mortality in patients with obstructive and nonobstructive CAD.

## Methods

### Study Population

**Figure 1:**
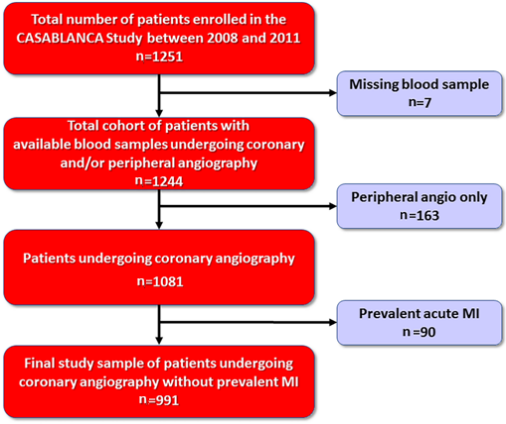
Study flow. Following removal of those without coronary angiography and those with prevalent myocardial infarction, the study sample comprised 991 patients. Angio indicates angiography; CASABLANCA, Catheter Sampled Blood Archive in Cardiovascular Diseases Study; MI, myocardial infarction.

All study procedures were approved by the Partners Healthcare institutional review board and consistent with the Declaration of Helsinki. The data, analytic methods, and study materials will not be made available to other researchers for purposes of reproducing the results or replicating the procedure. The CASABLANCA study was a prospective, single‐center, investigator‐initiated, observational cohort study undertaken at Massachusetts General Hospital in Boston.^13^ Over a 3‐year period between September 2008 and November 2011, 1251 participants undergoing coronary and peripheral angiography with or without intervention were enrolled. End point adjudication was performed using the guidance of the Third Universal Definition of Myocardial Infarction, with local fourth‐generation cardiac troponin T as the biomarker standard for diagnosis of MI; for the purposes of the adjudication, the 99th percentile cardiac troponin T concentration of 0.01 ng/mL was utilized. After excluding patients who underwent peripheral angiography only and patients with acute MI, our final study cohort for this analysis consisted of 991 patients (Figure 1).

After informed consent was obtained, demographics, clinical characteristics, reason for referral for angiography, and angiographic results (based on the final report from the procedure) were noted at the time of the procedure. As previously reported, patients were mainly referred for evaluation of CAD (Table S1).^13^

### Biomarker Testing

Immediately before angiography angiographic procedures, 15 mL of blood was obtained through a centrally placed vascular access sheath. All samples were promptly centrifuged for 10 minutes. Samples were aliquoted on ice and then immediately placed in a −80°C freezer for permanent storage.

Using aliquots that had undergone their first thaw, concentrations of hsTnI were measured using a single‐molecule counting assay (SMC troponin I; Singulex) performed on an Erenna platform. This very highly sensitive assay has a limit of detection of 0.5 ng/L and a 99th percentile reference limit of 6 ng/L in apparently healthy individuals.

### Follow‐up and Outcomes

Incident MI, heart failure (HF), cardiovascular death, and all‐cause mortality were recorded over a median follow‐up period of 1486 days (quartile 1: 1203 days; quartile 3: 1577 days). The mean follow‐up period was 1338 days (SD: 367 days). A detailed definition of each end point for CASABLANCA has been described previously.^13^ For identification of clinical end points during follow‐up, bioinformatics‐assisted review of medical records and phone follow‐up with patients and/or primary care physicians were performed. Each clinical end point was adjudicated by a panel of investigators. The Social Security Death Index and/or postings of death announcements were used to confirm status. No patients were lost to follow‐up.

For the purposes of the present analysis, obstructive CAD was defined as ≥70% stenosis in at least 1 vessel, and nonobstructive CAD was defined as stenosis >0% but <70%.

### Statistical Analysis

Baseline characteristics between those with hsTnl >99th versus <99th percentile were compared using the χ^2^ or Fisher exact test for dichotomous variables, the Cochran–Mantel–Haenszel test for categorical variables, and the *t* test or Kruskal–Wallis test for continuous variables. The following covariates were included in all cross‐sectional and prognostic analyses in addition to hsTnl: age, sex, heart rate, systolic blood pressure, diastolic blood pressure, atrial fibrillation or flutter, hypertension, CAD, chronic obstructive pulmonary disease, diabetes mellitus, chronic kidney disease, medications used (angiotensin‐converting enzyme inhibitor or angiotensin receptor blocker, beta blockers, calcium channel blocker, aldosterone antagonist, loop diuretics, nitrates), sodium, glomerular filtration rate, glucose, hemoglobin, myeloperoxidase, NT‐proBNP (N‐terminal pro‐B‐type natriuretic peptide), and cystatin C.

Logistic regression was first used to assess the cross‐sectional relationship between obstructive CAD and the above set of individual predictors. Covariates significant at 0.10 level were then included into a stepwise logistic regression, with hsTnl, age, and sex forced into the model.

Cox proportional hazards models were used to predict the following outcomes in all patients, in the subset of patients with obstructive CAD, and in the subset of patients with nonobstructive CAD, using the set of covariates listed above: incident MI, incident HF, cardiovascular death, all‐cause death, and the combined outcomes of incident MI, HF, and all‐cause death; incident MI and cardiovascular death; and incident MI and all‐cause death. Again, covariates significant at 0.10 level were then included in a stepwise Cox proportional hazards model, with hsTnl, age, and sex forced in. The analyses that included MI as an outcome were additionally adjusted for previous MI; all analyses that included HF as an outcome were additionally adjusted for previous HF.

Kaplan–Meier curves were compared using the log‐rank test. In all statistical analyses, a 2‐tailed *P* value of <0.05 was considered statistically significant. All analyses were performed using the SAS version 9.4 (SAS Institute).

## Results

### Baseline Characteristics

The median hsTnI concentration in the study sample was 4.19 ng/L (Table S2). Elevation in hsTnI ≥99th percentile was observed in 375 patients (38%). The most common reason for referral for coronary angiography was stable angina (44.9%). Other reasons for referral included chest pain (20.3%), unstable angina (13.5%), preoperative evaluation (13.2%), arrhythmia evaluation (6.7%), and transplant evaluation (1.2%).

Baseline characteristics of study participants, dichotomized as a function of elevated hsTnI ≥99th percentile, are detailed in Table 1. Those with versus without elevated hsTnI concentration were more likely to be older and typically had more complex medical histories; they were more likely to have prior history of diabetes mellitus, chronic kidney disease, peripheral arterial disease, HF, prior MI, atrial fibrillation or flutter, lower left ventricular ejection fraction, and higher right ventricular systolic pressure. In addition, those with elevated hsTnI typically had higher concentrations of other prognostic biomarkers (Table 1).

**Table 1:** Baseline Characteristics of Study Participants as a Function of hsTnI 99th Percentile

Characteristics	hsTnI <99th Percentile (n=616)	hsTnI ≥99th Percentile (n=375)	*P* Value
Demographic			
Age, y, mean±SD	65.03±10.79	68.98±11.83	<0.001
Male sex, %	69.97	75.20	0.08
Race, %			0.97
White	93.67	93.60	
Black	1.95	2.93	
Asian/Pacific	0.81	1.60	
Hispanic	2.11	1.87	
Vital signs, mean±SD			
Heart rate, beats/min	67.9±13.0	70.7±14.2	0.002
Systolic blood pressure, mm Hg	135.7±21.2	138.1±24.4	0.12
Diastolic blood pressure, mm Hg	72.5±11.0	73.1±12.1	0.41
Medical history, %			
Smoker	12.5	14.0	0.50
Atrial fibrillation/flutter	16.6	26.9	<0.001
Hypertension	71.3	77.3	0.04
CAD	50.0	53.9	0.24
Prior MI	21.4	28.0	0.02
HF	15.1	31.7	<0.001
Peripheral artery disease	14.5	23.5	<0.001
COPD	16.4	18.9	0.31
Diabetes mellitus type I/II	22.2	30.9	0.002
CVA/TIA	9.6	11.2	0.41
CKD	6.5	20.5	<0.001
Renal replacement therapy	1.1	6.7	<0.001
Prior angioplasty	9.6	11.7	0.28
Prior stent	27.9	27.5	0.88
Prior CABG	16.1	24.3	0.001
Medications, %			
ACEI/ARB	50.5	58.6	0.01
Beta blocker	72.6	68.2	0.14
Aldosterone antagonist	3.6	5.6	0.13
Loop diuretics	14.7	32.6	<0.001
Nitrates	19.1	18.5	0.80
CCB	22.5	26.5	0.15
Statin	70.4	72.2	0.55
Aspirin	76. 7	71.9	0.09
Warfarin	13.5	21.1	0.002
P2Y_12_ inhibitor	21.2	21.4	0.96
Echocardiography, mean±SD			
LVEF, %	59.8±13.4	50.5±17.6	<0.001
RSVP	37.7±10.0	45.0±11.6	<0.001
Laboratory measures, mean±SD			
Sodium, mmol/L	139.4±3.1	139.5±3.1	0.75
Blood urea nitrogen, mg/dL	19.0±8.0	24.0±12.5	<0.001
Creatinine, mg/dL	1.14±0.72	1.55±1.47	<0.001
Total cholesterol, mg/dL	152. 9±41.2	146.3±45.8	0.06
LDL cholesterol, mg/dL	83.4±32.7	79.3±36.7	0.16
Glycohemoglobin, %	6.35±1.60	6.64±1.23	0.14
Glucose, mg/dL	111.4±38.5	117.4±41.8	0.05
Hb, g/L	13.4±1.6	13.0±1.8	<0.001
Baseline biomarkers			
MPO, pmol/L, median (IQR)	391.2 (307.0–532.0)	455.8 (330.0–610.9)	<0.001
Troponin I, pg/mL, median (IQR)	4.1 (2.6–6.4)	28.7 (15.1–85.6)	<0.001
NT‐proBNP, pg/mL, median (IQR)	167.0 (71.0–462.0)	1088.0 (336.0–2789.0)	<0.001
Cystatin C, mg/L, median (IQR)	0.8 (0.7–0.9)	0.9 (0.7–1.2)	<0.001

ACEI indicates angiotensin‐converting enzyme inhibitor; ARB, angiotensin receptor blocker; CABG, coronary artery bypass grafting; CAD, coronary artery disease; CCB, calcium channel blocker; CKD, chronic kidney disease; COPD, chronic obstructive pulmonary disease; CVA, cerebrovascular accident; Hb, hemoglobin; HF, heart failure; hsTnI, high‐sensitivity troponin I; IQR, interquartile range; LDL, low‐density lipoprotein; LVEF, left ventricular ejection fraction; MI, myocardial infarction; MPO, myeloperoxidase; NT‐proBNP, N‐terminal pro‐B‐type natriuretic peptide; RVSP, right ventricular systolic pressure; TIA, transient ischemic attack.

### hsTnI and Coronary Angiography Results

Of the 991 patients included in this study, 619 patients were found to have obstructive CAD, 226 patients had nonobstructive CAD, and the remaining 146 patients either had no evidence of coronary disease or were missing information. Patients with an hsTnI ≥99th percentile had a higher degree and number of stenotic coronary lesions (Table 2). For example, 21.5% of patients without prevalent MI and hsTnI concentration ≥6 ng/L had severe (≥70% stenosis) 3‐vessel disease versus 14.7% without prevalent MI and hsTnI concentration <6 ng/L (*P*=0.006).

**Table 2:** Presence and Severity of CAD as a Function of hsTnI Concentrations Dichotomized Around the 99th Percentile Value

Angiography Results	hsTnI <99th Percentile (n=616), %	hsTnI ≥99th Percentile (n=375), %	*P* Value
≥30% coronary stenosis in ≥2 vessels	54.9	69.1	<0.001
≥30% coronary stenosis in ≥3 vessels	38.1	53.8	<0.001
≥50% coronary stenosis in ≥2 vessels	44.3	55.9	<0.001
≥50% coronary stenosis in ≥3 vessels	24.1	35.8	<0.001
≥70% coronary stenosis in ≥2 vessels	32.7	42.7	0.002
≥70% coronary stenosis in ≥3 vessels	14.7	21.5	0.006

Among patients without acute myocardial infarction, an elevated hsTnI was associated with more prevalent and extensive coronary stenosis. CAD indicates coronary artery disease; hsTnI, high‐sensitivity troponin I.

In multivariate analysis, dichotomized hsTnI (≥99th percentile) independently predicted obstructive CAD (odds ratio: 2.57; 95% confidence interval [CI], 1.73–3.80; *P*<0.001; Tables S3–S6). In receiver operating characteristic analyses, hsTnI had an area under the curve of 0.61 for obstructive CAD (95% CI, 0.58–0.65). A hsTnI concentration ≥6 ng/L had sensitivity of 44%, specificity of 72%, positive predictive value of 72%, and negative predictive value of 43% for obstructive CAD. An hsTnI concentration below the limit of detection had a negative predictive value of 37% for obstructive CAD (Table S7). Assessed as a continuous variable, log‐transformed hsTnI also independently predicted obstructive CAD (odds ratio: 1.43, *P*<0.001), yielding a C statistic of 0.82 (95% CI, 0.78–0.85).

### Prognostic Outcomes

**Figure 2:**
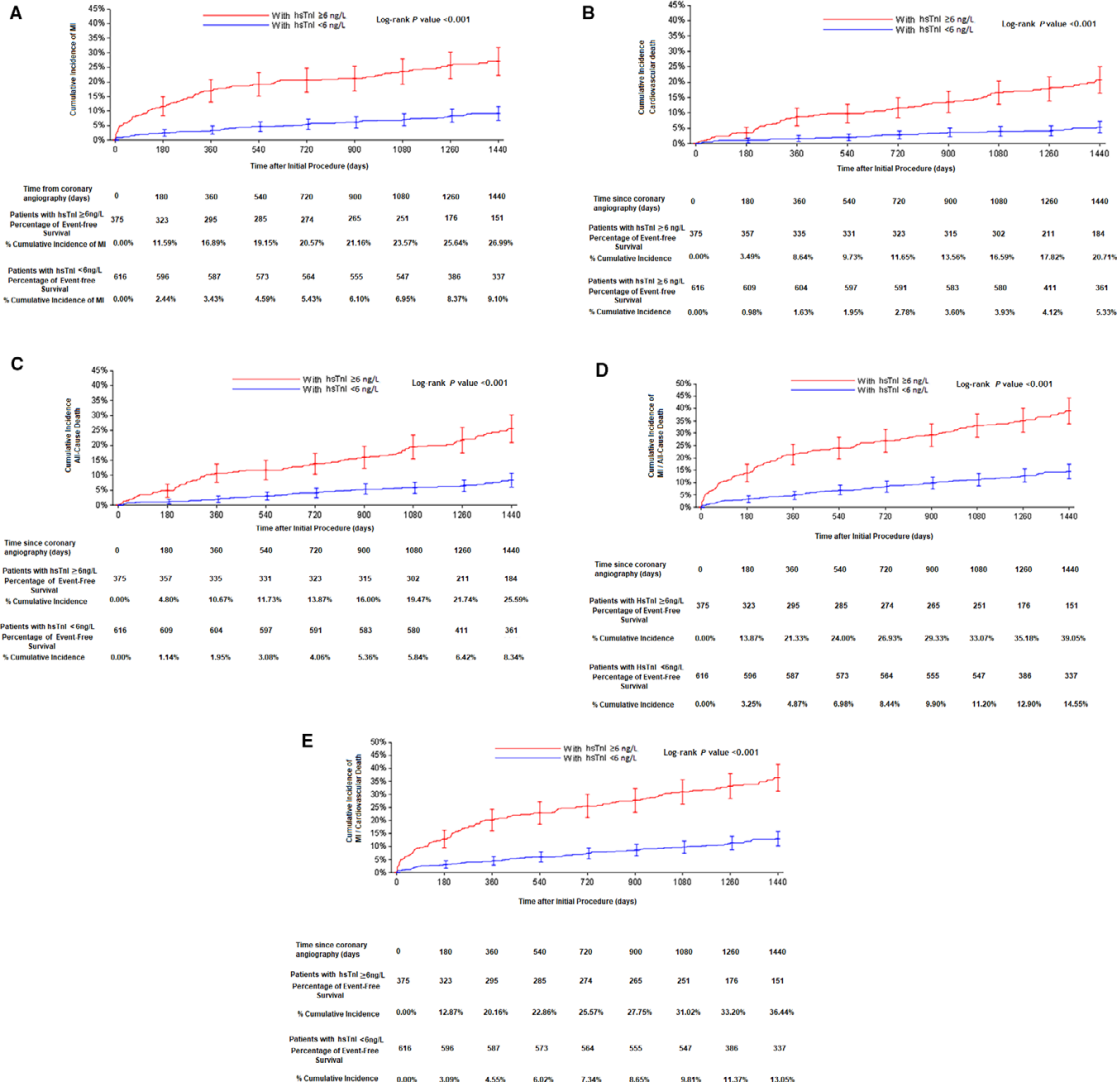
Cumulative event curves over a follow‐up period of 1440 days in patients with or without elevated hsTnI ≥6 ng/L for (A) incident MI, (B) incident cardiovascular death, (C) all‐cause death, (D) composite of MI and all‐cause death, and (E) composite of MI and cardiovascular death. hsTnI indicates high‐sensitivity cardiac troponin I; MI, myocardial infarction.

Over a median follow‐up period of 1486 days, there were 147 MIs, 238 HF exacerbations, 113 cardiovascular deaths, and 149 all‐cause deaths (Table S44). In Kaplan–Meier survival analyses, time to incident MI (Figure 2A) was substantially shorter and incidence was higher in those with hsTnI ≥99th percentile; the survival curves diverged early, and by end of follow‐up, cumulative incidence of MI was 27% versus 9% in those with elevated versus low hsTnI (log‐rank *P*<0.001). Similar patterns were seen relative to cardiovascular death (log‐rank *P*<0.001; Figure 2B), all‐cause death (log‐rank *P*<0.001; Figure 2C), the composite end point of MI and all‐cause death (log‐rank *P*<0.001; Figure 2D), and the composite end point of MI and cardiovascular death (log‐rank *P*<0.001; Figure 2E).

In fully adjusted multivariate analysis (Table 3), in all participants, hsTnI ≥99th percentile independently predicted incident MI (hazard ratio [HR]: 2.68; 95% CI, 1.86–3.85; *P*<0.001), incident HF (HR: 1.60; 95% CI, 1.21–2.13; *P*=0.01), cardiovascular death (HR: 2.29; 95% CI, 1.40–3.75; *P*=0.001), and all‐cause death (HR: 1.84; 95% CI, 1.22–2.78; *P*=0.004). In addition, an elevated hsTnI concentration independently predicted composite end points of incident MI, HF, and all‐cause mortality (HR: 1.62; *P*<0.001); incident MI and cardiovascular death (HR: 2.22; *P*<0.001), and incident MI and all‐cause death (HR: 2.13; *P*<0.001; Tables S8–S21). In a subanalysis of prognostic outcomes among men and women, hsTnI ≥99th percentile more strongly predicted all‐cause death in women (HR: 5.83; *P*<0.001) compared with men (HR: 1.48; *P*=0.10) and incident HF in men (HR: 1.71; *P*<0.001) compared with women (HR: 1.36; *P*=0.34). Similarly, hsTn ≥99th percentile predicted incident MI (HR: 2.60; *P*<0.001 in men; HR: 2.61; *P*=0.01 in women) and cardiovascular death (HR: 2.28; *P*=0.004 in men; HR: 2.21; *P*=0.07 in women; Tables S22–S29).

**Table 3:** Prognostic Outcomes as a Function of hsTnI ≥99th Percentile in All Participants and in Those With Presence of Obstructive CAD (Defined as ≥70% Stenosis)

Outcome	HR	95% CI	*P* Value
Incident MI			
All participants (N=991)	2.68	1.86–3.85	<0.001
Obstructive CAD (n=619)	1.87	1.16–3.00	0.01
Nonobstructive CAD (N=226)	8.41	2.77–25.5	<0.001
Incident HF			
All participants (N=991)	1.60	1.21–2.13	0.001
Obstructive CAD (n=619)	1.17	0.76–1.80	0.46
Nonobstructive CAD (n=226)	1.61	0.85–3.06	0.14
Cardiovascular death			
All participants (N=991)	2.29	1.40–3.75	0.001
Obstructive CAD (n=619)	2.74	1.48–5.06	0.001
Nonobstructive CAD (n=226)	3.60	1.15–11.3	0.03
All‐cause death			
All participants (N=991)	1.84	1.22–2.78	0.004
Obstructive CAD (n=619)	2.06	1.26–3.37	0.004
Nonobstructive CAD (n=226)	2.25	0.83–6.12	0.11
Incident MI/HF/all‐cause death			
All participants (N=991)	1.62	1.26–2.08	<0.001
Obstructive CAD (n=619)	1.71	1.25–2.33	<0.001
Nonobstructive CAD (n=226)	1.60	0.88–2.90	0.12
Incident MI/cardiovascular death			
All participants (N=991)	2.22	1.60–3.08	<0.001
Obstructive CAD (n=619)	2.06	1.40–3.05	<0.001
Nonobstructive CAD (n=226)	4.70	2.17–10.18	<0.001
Incident MI/all‐cause death			
All participants (N=991)	2.13	1.56–2.90	<0.001
Obstructive CAD (n=619)	2.06	1.42–2.98	<0.001
Nonobstructive CAD (n=226)	3.62	1.78–7.37	<0.001

CAD indicates coronary artery disease; CI, confidence interval; HF, heart failure; HR, hazard ratio; hsTnI, high‐sensitivity troponin I; MI, myocardial infarction.

In an effort to better understand the prognostic meaning of hsTnI concentrations in those with obstructive versus nonobstructive CAD, subanalyses were undertaken by partitioning patients based on their angiographic results (Table 3). In patients with ≥70% coronary stenosis (n=619), hsTnI ≥99th percentile independently predicted incident MI (HR: 1.87; *P*=0.01; Figures S1 and S2), cardiovascular mortality (HR: 2.74; *P*=0.001), and the composite end point of MI and all‐cause death (HR: 2.06; *P*<0.001). In patients with nonobstructive CAD (n=226), hsTnI ≥99th percentile even more strongly predicted incident MI (HR: 8.41; *P*<0.001; Figures S2 and S3), cardiovascular mortality (HR: 3.60; *P*=0.03), and the composite end point of MI and all‐cause death (HR: 3.62; *P*<0.001; Tables S30–S43).

## Discussion

In a large prospective cohort study of patients without prevalent MI undergoing diagnostic coronary angiography, we found that elevations in hsTnI were common, were associated with a higher prevalence of obstructive CAD, and independently predicted numerous cardiovascular events including incident MI, incident HF, cardiovascular death, and all‐cause death. Risk associated with elevated hsTnI appeared early and was sustained across follow‐up. Furthermore, we found that elevated hsTnI remained prognostic for adverse outcome regardless of degree of coronary obstruction and appeared even more prognostic for those without obstructive CAD.

Our observations that hsTn concentrations correlated with the number and degree of stenotic coronary lesions and that elevated hsTn is an independent predictor of obstructive CAD lend important understanding of how such hsTn values should be interpreted clinically. In a prior case–control study involving 904 patients with stable CAD and 412 patients with chest pain but without significant CAD on coronary angiogram, Ndrepepa et al demonstrated hsTnT concentrations correlated with an increasing number of stenotic coronary lesions and with 30% higher likelihood of the presence of CAD.^14^ Such case–control methods may underestimate the predictive value of hsTn in part because of the inability to easily mimic pretest probability of a patient population referred for diagnostic coronary angiography. Our results generated with a substantially higher sensitivity hsTnI method in a population with higher pretest probability suggest that concentrations of hsTnI even more strongly predict presence of obstructive CAD. Lending context to our findings, among a lower risk population of patients with acute chest pain, we previously showed that very low hsTnI concentrations (measured with another assay) excluded prevalent CAD with higher negative predictive value.^15^ Taken together, our results suggest that among patients with lower pretest probability, very low concentrations of hsTn may confidently exclude CAD, whereas in those with higher pretest probability, the greater value of hsTn may identify obstructive CAD. These results complement those of Twerenbold et al, who found that logical ordering and interpretation of hsTn concentrations improved accuracy of diagnostic coronary angiography.^16^ Given the present release of hsTn methods in the United States, it is imperative that clinicians understand the impact of pretest probability when ordering and interpreting hsTn results.

Notably, we found hsTn concentrations ≥99th percentile to be a robust independent predictor of all‐cause mortality, cardiovascular death, incident MI, and incident HF. Interestingly, in patients with nonobstructive CAD, we found that an elevated hsTnI concentration was a powerful predictor of incident MI (HR: 8.41; *P*<0.001) and cardiovascular mortality (HR: 3.60; *P*=0.03). This is a cohort that is often overlooked in clinical practice when discovered to have nonobstructive disease; however, over a 1486‐day follow‐up period, our study found that the cumulative incidence of MI was actually higher in patients with nonobstructive disease and elevated hsTnI ≥99th percentile (23%) compared with patients with obstructive CAD and an hsTnI concentration <99th percentile (12%); the incidence of incident MI in patients with obstructive CAD and hsTnI ≥99th percentile was 30%. Further studies should evaluate the utility of hsTnI to trigger application of more aggressive medical therapies to reduce atherothrombotic risk (eg, directed application of higher dose statin therapy, PCSK9 [proprotein convertase subtilisin/kexin type 9] inhibitor therapy, direct oral anticoagulant use) in this cohort.

Although this study is one of the first of its kind to explore diagnostic and prognostic meanings of a novel single‐molecule hsTnI method, it has limitations. We excluded those patients with prevalent MI at the time of enrollment (judged using cardiac troponin T at its 99th percentile); however, it is possible that some patients would have been recognized as having MI if adjudicated with the hsTnI under study in the present analysis. This issue of equipoise is an inevitable consequence of higher sensitivity, and it is fair to expect such limitations to affect any study examining hsTn methods in patients with ischemic heart disease. Concentrations of hsTnI were measured at a single point in time at the time of angiography and may not reflect levels at future time periods. It is necessary to note the hsTnI assay used in this analysis is time‐consuming compared with processing of conventional troponin assays; more efficient versions of such highly sensitive methods would be needed to translate these results for efficient clinical use. Our results need further validation and should not be extrapolated to the general population without suspected CAD because these patients were not included in our study. Whether aggressive treatment strategies in patients with an elevated hsTn concentration would modify their risk of future cardiovascular events remains unknown and requires further investigation.

With assays of increasing sensitivity coming into more widespread use, clinicians face detection of abnormal values in patients not thought to have acute MI. This has led to increased use of nonspecific terms such as *myocardial injury* that lack therapeutic imperatives. Our results suggest even slight elevation in hsTnI concentrations predicted the presence of obstructive CAD and independently predicted major cardiovascular events. Our results add novel and substantial clinical context to hsTnI concentrations in a commonly encountered patient population (those sent for angiography) while setting the stage for analyses of how hsTn may be leveraged in clinical trials of therapeutic intervention for such patients.

## Conclusions

HsTnI is a powerful predictor of the presence of CAD in patients sent for angiography not thought to have acute MI. In addition, hsTnI predicts incident cardiovascular events in patients with either obstructive or nonobstructive CAD. Our results will help to clarify the meaning of elevated hsTn in patients without MI. Further studies should evaluate the utility of hsTnI to trigger application of more aggressive medical therapies to reduce atherothrombotic risk (eg, more aggressive lipid lowering, use of direct oral anticoagulants) in this cohort.

## Sources of Funding

Dr Ibrahim is supported by the Dennis and Marilyn Barry Fellowship in cardiology research. Dr Januzzi is supported in part by the Hutter Family Professorship in Cardiology. Dr Gaggin is supported in part by the Ruth and James Clark Fund for Cardiac Research Innovation. This work was supported by a grant from Singulex.

## Disclosures

Dr Januzzi has received grant support from Roche Diagnostics, Siemens, Cleveland Heart Labs and Prevencio, consulting income from Roche Diagnostics, Critical Diagnostics, Philips, and Novartis, and participates in clinical end point committees/data safety monitoring boards for Abbvie, Bayer, Pfizer, Novartis, Amgen, Janssen, and Boehringer Ingelheim. Dr Gaggin has received grant support from Roche and Portola; consulting income from Roche Diagnostics, American Regent, Amgen, Boston Heart Diagnostics and Critical Diagnostics; research payments for clinical end point committees for EchoSense. The remaining authors have nothing to disclose. All authors have approved the final article.

## Supporting information


**Table S1.** Reason for Referral for Angiography for the 991 Patients Included in the Study
**Table S2.** Baseline High‐Sensitivity Troponin I Concentrations Among All Patients and Subgroups Stratified by Coronary Artery Disease Status
**Table S3.** Multivariate Logistic Regression on Obstructive Coronary Artery Disease (≥70% Stenosis) With Age, Sex, Dichotomized Singulex High‐Sensitivity Troponin I, and Stepwise Selection on Baseline Biomarkers* and Other Baseline Covariates** (Full Study Cohort, N=991)
**Table S4.** Multivariate Logistic Regression on Obstructive Coronary Artery Disease (≥70% Stenosis) With Age, Sex, Continuous High‐Sensitivity Troponin I, and Stepwise Selection on Baseline Biomarkers* and Other Baseline Covariates** (Full Study Cohort, N=991)
**Table S5.** Multivariate Logistic Regression on Obstructive Coronary Artery Disease (≥70% stenosis), With Age, Sex and Natural‐Log Transformed Continuous Singulex High‐Sensitivity Troponin and Baseline Biomarkers* and Other Covariates** (Full Study Cohort, N=991)
**Table S6.** Multivariate Logistic Regression on Obstructive Coronary Artery Disease (≥70% Stenosis) With Age, Sex, Dichotomized Singulex High‐Sensitivity Troponin I, and Stepwise Selection on Baseline Biomarkers and Other Baseline Covariates (Full Cohort Excluding Patients With Unstable Angina, n=857)
**Table S7.** Negative Predictive Value for Obstructive Coronary Artery Disease by Concentration of High‐Sensitivity Troponin I
**Table S8.** Multivariate Cox Regression on Incident Myocardial Infarction With Age, Sex, Dichotomized Singulex High‐Sensitivity Troponin I, and Stepwise Selection on Baseline Biomarkers* and Other Baseline Covariates** (Full Study Cohort, N=991)
**Table S9.** Multivariate Cox Regression on Incident Myocardial Infarction With Age, Sex, Dichotomized Singulex High‐Sensitivity Troponin I, and Stepwise Selection on Baseline Biomarkers* and Other Baseline Covariates** (Full Cohort Excluding Patients With Unstable Angina, n=857)
**Table S10.** Multivariate Cox Regression on Incident Heart Failure With Age, Sex, Dichotomized Singulex High‐Sensitivity Troponin I, and Stepwise Selection on Baseline Biomarkers* and Other Baseline Covariates** (Full Study Cohort, N=991)
**Table S11.** Multivariate Cox Regression on Incident Heart Failure With Age, Sex, Dichotomized Singulex High‐Sensitivity Troponin I, and Stepwise Selection on Baseline Biomarkers* and Other Baseline Covariates** (Full Cohort Excluding Patients With Unstable Angina, n=857)
**Table S12.** Multivariate Cox Regression on Cardiovascular Death With Age, Sex, Dichotomized Singulex High‐Sensitivity Troponin I, and Stepwise Selection on Baseline Biomarkers and Other Baseline Covariates (Full Study Cohort, N=991)
**Table S13.** Multivariate Cox Regression on Cardiovascular Death With Age, Sex, Dichotomized Singulex High‐Sensitivity Troponin I, and Stepwise Selection on Baseline Biomarkers* and Other Baseline Covariates** (Full Cohort Excluding Patients With Unstable Angina, n=857)
**Table S14.** Multivariate Cox Regression on All‐Cause Death With Age, Sex, Dichotomized Singulex High‐Sensitivity Troponin I, and Stepwise Selection on Baseline Biomarkers* and Other Baseline Covariates** (Full Study Cohort, N=991)
**Table S15.** Multivariate Cox Regression on All‐Cause Death With Age, Sex, Dichotomized Singulex High‐Sensitivity Troponin I, and Stepwise Selection on Baseline Biomarkers* and Other Baseline Covariates** (Full Cohort Excluding Patients With Unstable Angina, n=857)
**Table S16.** Multivariate Cox Regression on Composite End Point of Myocardial Infarction, Heart Failure, and All‐Cause Death With Age, Sex, Dichotomized Singulex High‐Sensitivity Troponin I, and Stepwise Selection on Baseline Biomarkers and Other Baseline Covariates (Full Study Cohort, N=991)
**Table S17.** Multivariate Cox Regression on Composite End Point of Myocardial Infarction, Heart Failure, and All‐Cause Death With Age, Sex, Dichotomized Singulex High‐Sensitivity Troponin I, and Stepwise Selection on Baseline Biomarkers* and Other Baseline Covariates** (Full Cohort Excluding Patients With Unstable Angina, n=857)
**Table S18.** Multivariate Cox Regression on Composite End Point of Myocardial Infarction and Cardiovascular Death With Age, Sex, Dichotomized Singulex High‐Sensitivity Troponin I, and Stepwise Selection on Baseline Biomarkers* and Other Baseline Covariates** (Full Study Cohort, N=991)
**Table S19.** Multivariate Cox Regression on Composite End Point of Myocardial Infarction and Cardiovascular Death With Age, Sex, Dichotomized Singulex High‐Sensitivity Troponin I, and Stepwise Selection on Baseline Biomarkers* and Other Baseline Covariates** (Full Cohort Excluding Patients With Unstable Angina, n=857)
**Table S20.** Multivariate Cox Regression on Composite End Point of Myocardial Infarction and All‐Cause Death With Age, Sex, Dichotomized Singulex High‐Sensitivity Troponin I, and Stepwise Selection on Baseline Biomarkers* and Other Baseline Covariates** (Full Study Cohort, N=991)
**Table S21.** Multivariate Cox Regression on Composite End Point of Myocardial Infarction and All‐Cause Death With Age, Sex, Dichotomized Singulex High‐Sensitivity Troponin I, and Stepwise Selection on Baseline Biomarkers* and Other Baseline Covariates** (Full Cohort Excluding Patients With Unstable Angina, n=857)
**Table S22.** Multivariate Cox Proportional Hazards Regression on Incident Myocardial Infarction With Age, Sex, Dichotomized Singulex High‐Sensitivity Troponin I, and Other Covariates* (Male, n=713)
**Table S23.** Multivariate Cox Proportional Hazards Regression on Incident Myocardial Infarction With Age, Sex, Dichotomized Singulex High‐Sensitivity Troponin I, and Other Covariates* (Female, n=278)
**Table S24.** Multivariate Cox Proportional Hazards Regression on Incident Congestive Heart Failure With Age, Sex, Dichotomized Singulex High‐Sensitivity Troponin I, and Other Covariates* Forced Into the Model (Male, n=713)
**Table S25.** Multivariate Cox Proportional Hazards Regression on Incident Congestive Heart Failure With Age, Sex, Dichotomized Singulex High‐Sensitivity Troponin I, and Other Covariates* (Female, n=278)
**Table S26.** Multivariate Cox Proportional Hazards Regression on Cardiovascular Death With Age, Sex, Dichotomized Singulex High‐Sensitivity Troponin I, and Other Covariates* (Male, n=713)
**Table S27.** Multivariate Cox Proportional Hazards Regression on Cardiovascular Death With Age, Sex, Dichotomized Singulex High‐Sensitivity Troponin I, and Other Covariates* (Female, n=278)
**Table S28.** Multivariate Cox Proportional Hazards Regression on All‐Cause Death With Age, Sex, Dichotomized Singulex High‐Sensitivity Troponin I, and Other Covariates* (Male, n=713)
**Table S29.** Multivariate Cox Proportional Hazards Regression on All‐Cause Death With Age, Sex, Dichotomized Singulex High‐Sensitivity Troponin I, and Other Covariates* (Female, n=278)
**Table S30.** Multivariate Cox Proportional Hazards Regression on Incident Myocardial Infarction With Age, Sex, Dichotomized Singulex High‐Sensitivity Troponin I, and Other Covariates* (Patients With Obstructive Coronary Artery Disease Only, n=619)
**Table S31.** Multivariate Cox Proportional Hazards Regression on Cardiovascular Mortality With Age, Sex, Dichotomized Singulex High‐Sensitivity Troponin I, and Other Covariates* (Patients With Obstructive Coronary Artery Disease Only, n=619)
**Table S32.** Multivariate Cox Proportional Hazards Regression on Incident Congestive Heart Failure With Age, Sex, Dichotomized Singulex High‐Sensitivity Troponin I, and Other Covariates* (Patients With Obstructive Coronary Artery Disease Only, n=619)
**Table S33.** Multivariate Cox Proportional Hazards Regression on All‐Cause Mortality With Age, Sex, Dichotomized Singulex High‐Sensitivity Troponin I, and Other Covariates* (Patients With Obstructive Coronary Artery Disease Only, n=619)
**Table S34.** Multivariate Cox Proportional Hazards Regression on Incident Heart Failure, Myocardial Infarction, and All‐Cause Death, With Age, Sex, Dichotomized Singulex High‐Sensitivity Troponin I, and Other Covariates* (Patients With Obstructive Coronary Artery Disease Only, n=619)
**Table S35.** Multivariate Cox Proportional Hazards Regression on Composite Outcome of Myocardial Infarction and All‐Cause Death, With Age, Sex, Dichotomized Singulex High‐Sensitivity Troponin I, and Other Covariates* (Patients With Obstructive Coronary Artery Disease Only, n=619)
**Table S36.** Multivariate Cox Proportional Hazards Regression on Composite Outcome of Myocardial Infarction and Cardiovascular Death, With Age, Sex, Dichotomized Singulex High‐Sensitivity Troponin I, and Other Covariates* (Patients With Obstructive Coronary Artery Disease Only, n=619)
**Table S37.** Multivariate Cox Proportional Hazards Regression on Incident Myocardial Infarction With Age, Sex, Dichotomized Singulex hsTnl and Other Covariates* (Patients With Nonobstructive Coronary Artery Disease Only, n=226)
**Table S38.** Cox Proportional Hazards Regression on Incident Congestive Heart Failure With Age, Sex, Dichotomized Singulex High‐Sensitivity Troponin I, and Other Covariates* (Patients With Nonobstructive Coronary Artery Disease, n=226)
**Table S39.** Multivariate Cox Proportional Hazards Regression on Cardiovascular Mortality With Age, Sex, Dichotomized Singulex High‐Sensitivity Troponin I, and Other Covariates* (Patients With Nonobstructive Coronary Artery Disease Only, n=226)
**Table S40.** Multivariate Cox Proportional Hazards Regression on All‐Cause Mortality With Age, Sex, Dichotomized Singulex High‐Sensitivity Troponin I, and Other Covariates* (Patients With Nonobstructive Coronary Artery Disease Only, n=226)
**Table S41.** Multivariate Cox Proportional Hazards Regression on Incident Heart Failure, Myocardial Infarction, and Death, With Age, Sex, Dichotomized Singulex High‐Sensitivity Troponin I, and Other Covariates* (Patients With Nonobstructive Coronary Artery Disease Only, n=226)
**Table S42.** Multivariate Cox Proportional Hazards Regression on Composite Outcome of Myocardial Infarction and All‐Cause Death, With Age, Sex, Dichotomized Singulex High‐Sensitivity Troponin I, and Other Covariates* (Patients With Nonobstructive Coronary Artery Disease Only, n=226)
**Table S43.** Multivariate Cox Proportional Hazards Regression on Composite Outcome of Myocardial Infarction and Cardiovascular Death, With Age, Sex, Dichotomized Singulex High‐Sensitivity Troponin I, and Other Covariates (Patients With Nonobstructive Coronary Artery Disease Only, n=226)
**Table S44.** Proportionality Test Result for Myocardial Infarction (MI); Heart Failure (HF); Cardiovascular Death; MI and Cardiovascular Death; MI and All‐Cause Death; and HF, MI, and All‐Cause Death (Full Cohort, N=991)
**Figure S1.** Kaplan–Meier curve for cumulative incidence of myocardial infarction with obstructive coronary artery disease (≥70% stenosis).
**Figure S2.** Kaplan–Meier curve for cumulative incidence of myocardial infarction with obstructive (≥70% stenosis) vs nonobstructive coronary artery disease (<70% but >0% stenosis).
**Figure S3.** Kaplan–Meier curve for cumulative incidence of myocardial infarction with nonobstructive coronary artery disease (<70% but >0% stenosis).
