# Supplementary material for: Single‐Molecule Counting of High‐Sensitivity Troponin I in Patients Referred for Diagnostic Angiography: Results From the CASABLANCA (Catheter Sampled Blood Archive in Cardiovascular Diseases) Study
Source: J Am Heart Assoc. 2018 Mar 8;7(6):e007975. doi: 10.1161/JAHA.117.007975 (PMC5907559; doi:10.1161/JAHA.117.007975)
Supplement: Supplementary file 1 — Table S1. Reason for Referral for Angiography for the 991 Patients Included in the Study Table S2. Baseline High‐Sensitivity Troponin I Concentrations Among All Patients and Subgroups Stratified by Coronary Artery Disease Status Table S3. Multivariate Logistic Regression on Obstructive Coronary Artery Disease (≥70% Stenosis) With Age, Sex, Dichotomized Singulex High‐Sensitivity Troponin I, and Stepwise Selection on Baseline Biomarkers* and Other Baseline Covariates** (Full Study Cohort, N=991) Table S4. Multivariate Logistic Regression on Obstructive Coronary Artery Disease (≥70% Stenosis) With Age, Sex, Continuous High‐Sensitivity Troponin I, and Stepwise Selection on Baseline Biomarkers* and Other Baseline Covariates** (Full Study Cohort, N=991) Table S5. Multivariate Logistic Regression on Obstructive Coronary Artery Disease (≥70% stenosis), With Age, Sex and Natural‐Log Transformed Continuous Singulex High‐Sensitivity Troponin and Baseline Biomarkers* and Other Covariates** (Full Study Cohort, N=991) Table S6. Multivariate Logistic Regression on Obstructive Coronary Artery Disease (≥70% Stenosis) With Age, Sex, Dichotomized Singulex High‐Sensitivity Troponin I, and Stepwise Selection on Baseline Biomarkers and Other Baseline Covariates (Full Cohort Excluding Patients With Unstable Angina, n=857) Table S7. Negative Predictive Value for Obstructive Coronary Artery Disease by Concentration of High‐Sensitivity Troponin I Table S8. Multivariate Cox Regression on Incident Myocardial Infarction With Age, Sex, Dichotomized Singulex High‐Sensitivity Troponin I, and Stepwise Selection on Baseline Biomarkers* and Other Baseline Covariates** (Full Study Cohort, N=991) Table S9. Multivariate Cox Regression on Incident Myocardial Infarction With Age, Sex, Dichotomized Singulex High‐Sensitivity Troponin I, and Stepwise Selection on Baseline Biomarkers* and Other Baseline Covariates** (Full Cohort Excluding Patients With Unstable Angina, n=857) Table S10. Multivariate Cox Regre [file JAH3-7-e007975-s001.pdf]

SUPPLEMENTAL MATERIAL

Table S1. Reason for referral for angiography for the 991 patients included in the study.

Characteristic	Subjects (N = 991)
Unstable angina	13.52% (134/991)
Stable angina	44.90% (445/991)
Chest pain	20.28% (201/991)
Arrhythmia evaluation	6.67% (66/989)
Transplant coronary evaluation	1.21% (12/991)
Preoperative evaluation	13.22% (131/991)

N=number of patients.

Table S2. Baseline hsTnI concentrations among all patients and subgroups stratified by CAD status.

.	**Obstructive CAD (N = 619)**	**Non-obstructive CAD (N = 226)**	**No CAD (N = 146)**	**All (N = 991)**
**High-sensitivity troponin I**				
Mean ± SD (N)	87.46±746.20 (619)	16.72±75.98 (226)	10.84±31.41 (146)	60.04±591.86 (991)
Median (Q1, Q3)	5.02 (2.37,12.50)	3.38 (1.95,7.37)	3.33 (1.54,6.56)	4.19 (2.09,10.11)
Range (min, max)	(0.26,16521.66)	(0.33,960.71)	(0.05,220.75)	(0.05,16521.66)
**HsTnl ≥99th percentile (ng/L)**	43.46% (269/619)	29.20% (66/226)	27.40% (40/146)	37.84% (375/991)

CAD= coronary artery disease, HsTnI= high sensitivity troponin I, N= number of patients, Q= quartile, SD= standard deviation.

Table S3. Multivariate Logistic Regression on Obstructive CAD (≥70% Stenosis) with Age, Sex, Dichotomized Singulex hsTnl and Stepwise Selection on Baseline Biomarkers*, and other Baseline Covariates.**
Full study cohort (N=991).

Characteristics	Parameter Estimate	Standard Error	Odds Ratio	95% CI	P-Value
Age, years	0.00821	0.00861	1.01	[0.99, 1.03]	0.34
Male sex	1.4407	0.2229	4.22	[2.73, 6.54]	<0.001
hsTnI ≥99th percentile, ng/L	0.9430	0.2005	2.57	[1.73, 3.80]	<0.001
Nitrates	0.9401	0.2810	2.56	[1.48, 4.44]	<0.001
Sodium, mmol/L	-0.0699	0.0300	0.93	[0.88, 0.99]	0.02
Diabetes mellitus	0.5576	0.2248	1.75	[1.12, 2.71]	0.01
Coronary artery disease	1.2461	0.1942	3.48	[2.38, 5.09]	<0.001
Atrial Fibrillation/Flutter	-0.7879	0.2292	0.46	[0.29, 0.71]	<0.001
Heart Rate (Beat/min)	-0.0175	0.00656	0.98	[0.97, 1.00]	0.008
Hemoglobin, g/L	-0.1842	0.0624	0.83	[0.74, 0.94]	0.003
Beta blocker	0.5898	0.2000	1.80	[1.22, 2.67]	0.003

CI= confidence interval, hsTnI= high sensitivity troponin I

*Baseline Biomarkers include: myeloperoxidase, NT-proBNP= amino-terminal pro-B type natriuretic peptide, Cystatin C.
**Other Covariates include: Heart Rate, Systolic Blood Pressure, Diastolic Blood Pressure, Atrial Fibrillation/Flutter, Hypertension, Coronary Artery Disease, Heart Failure, COPD, Diabetes type I/type II, chronic kidney disease, ACE-I/ARB, Beta Blocker, Aldosterone Antagonist, Loop Diuretics, Nitrates, calcium channel blocker, Sodium, eGFR (MDRD), Glucose, and hemoglobin.

Table S4. Multivariate Logistic Regression on Obstructive CAD (≥70% Stenosis) with Age, Sex, Continuous hsTnl and Stepwise Selection on Baseline Biomarkers*, and other Baseline Covariates.**
Full study cohort (N=991).

Characteristics	Parameter Estimate	Standard Error	P-Value	Odds Ratio	95% CI
Age, years	0.0148	0.00832	0.08	1.02	[1.00, 1.03]
Male sex	1.5750	0.2171	<0.001	4.83	[3.16, 7.39]
Continuous hsTnl, ng/L	0.00281	0.00121	0.02	1.00	[1.00, 1.01]
Nitrates	0.8378	0.2697	0.002	2.31	[1.36, 3.92]
Diabetes mellitus	0.6508	0.2184	0.003	1.92	[1.25, 2.94]
Coronary Artery Disease	1.2124	0.1877	<0.001	3.36	[2.33, 4.86]
Atrial Fibrillation/Flutter	-0.6856	0.2221	0.002	0.50	[0.33, 0.78]
Heart Rate (Beat/min)	-0.0144	0.00634	0.02	0.99	[0.97, 1.00]
Hemoglobin, g/L	-0.2174	0.0600	<0.001	0.81	[0.72, 0.90]
Beta blocker	0.5514	0.1938	0.004	1.74	[1.19, 2.54]

CI= confidence interval, hsTnI= high sensitivity troponin I.
*Baseline Biomarkers include: myeloperoxidase, NT-proBNP= amino-terminal pro-B type natriuretic peptide, Cystatin C.
**Other Covariates include: Heart Rate, Systolic Blood Pressure, Diastolic Blood Pressure, Atrial Fibrillation/Flutter, Hypertension, Coronary Artery Disease, Heart Failure, COPD, Diabetes type I/type II, chronic kidney disease, ACE-I/ARB, Beta Blocker, Aldosterone Antagonist, Loop Diuretics, Nitrates, calcium channel blocker, Sodium, eGFR (MDRD), Glucose, and hemoglobin.

Table S5. Multivariate Logistic Regression on Obstructive CAD (≥70% stenosis), with age, sex and Natural-Log Transformed continuous Singulex High Sensitivity Troponin and baseline biomarkers* and other covariates**
Full study cohort (N=991).

Characteristics	Parameter Estimate	Standard Error	P-Value	Odds Ratio	95% CI
Age, years	0.00728	0.00873	0.40	1.007	[0.99, 1.02]
Male sex	1.4244	0.2249	<0.001	4.16	[2.67, 6.46]
Log Singulex High Sensitivity Troponin, ng/L	0.3596	0.0704	<0.001	1.43	[1.25, 1.64]
Nitrates	0.9621	0.2825	<0.001	2.62	[1.50, 4.55]
Sodium, mmol/L	-0.0620	0.0300	0.04	0.94	[0.89, 1.00]
Diabetes mellitus	0.5445	0.2259	0.02	1.72	[1.11, 2.68]
Coronary Artery Disease	1.2446	0.1945	<0.001	3.47	[2.37, 5.08]
Atrial Fibrillation/Flutter	-0.7566	0.2284	<0.001	0.47	[0.30, 0.73]
Heart Rate (Beat/min)	-0.0176	0.00655	0.007	0.98	[0.97, 1.00]
Hemoglobin, g/L	-0.1887	0.0630	0.003	0.83	[0.73, 0.94]
Beta blocker	0.5586	0.2009	0.005	1.75	[1.18, 2.59]

CI= confidence interval, Hgb= hemoglobin, hsTnI= high sensitivity troponin I.

*Baseline Biomarkers include: myeloperoxidase, NT-proBNP= amino-terminal pro-B type natriuretic peptide, Cystatin C.
** Other Covariates: Heart Rate, Systolic Blood Pressure, Diastolic Blood Pressure, Atrial Fibrillation/Flutter, Hypertension, Coronary Artery Disease, Heart Failure, COPD, Diabetes type I/type II, CKD, ACE-I/ARB, Beta Blocker, Aldosterone Antagonist, Loop Diuretics, Nitrates, CCB, Sodium, GFR(MDRD), Glucose, and HGB.
Stepwise selection: levels for entry into and staying in the model are both 0.1.

Table S6. Multivariate Logistic Regression on Obstructive CAD (>=70% Stenosis) with Age, Sex, Dichotomized Singulex hsTnl and Stepwise Selection on Baseline Biomarkers and other Baseline Covariates.
Full cohort excluding patients with unstable angina (N=857)

Characteristics	Parameter Estimate	Standard Error	P-Value	Odds Ratio	95% CI
Age, years	0.00956	0.00890	0.28	1.01	[0.99, 1.03]
Male sex	1.5107	0.2408	<0.001	4.53	[2.83, 7.26]
hsTnI ≥99th percentile	0.7257	0.2121	0.001	2.07	[1.36, 3.13]
Nitrates	1.1081	0.3243	0.001	3.03	[1.60, 5.72]
Sodium, mmol/L	-0.0775	0.0317	0.01	0.93	[0.87, 0.98]
Diabetes mellitus	0.6336	0.2346	0.007	1.88	[1.19, 2.98]
Coronary Artery Disease	1.1889	0.2059	<0.001	3.28	[2.19, 4.92]
Atrial Fibrillation/Flutter	-0.7497	0.2390	0.002	0.47	[0.30, 0.75]
Heart Rate (Beat/min)	-0.0111	0.00686	0.11	0.99	[0.98, 1.00]
Hemoglobin, g/L	-0.1838	0.0656	0.005	0.83	[0.73, 0.95]
Beta blocker	0.6024	0.2089	0.004	1.83	[1.21, 2.75]

CI= confidence interval, hsTnI= high sensitivity troponin I.
*Baseline Biomarkers include: myeloperoxidase, NT-proBNP= amino-terminal pro-B type natriuretic peptide, Cystatin C.
**Other Covariates include: Heart Rate, Systolic Blood Pressure, Diastolic Blood Pressure, Atrial Fibrillation/Flutter, Hypertension, Coronary Artery Disease, Heart Failure, COPD, Diabetes type I/type II, chronic kidney disease, ACE-I/ARB, Beta Blocker, Aldosterone Antagonist, Loop Diuretics, Nitrates, calcium channel blocker, Sodium, eGFR (MDRD), Glucose, and hemoglobin

Table S7. Negative predictive value for obstructive CAD by concentration of hsTnI.

hsTnI concentration (ng/L)	Number of patients	Number of correctly predicted non-event*	Number of false predicted non-event	Negative predictive value
<0.5	16	356	614	0.37
<1.0	64	323	599	0.35
<1.5	133	292	561	0.34
<2.0	222	256	508	0.34
<2.5	311	223	453	0.33
<3.0	371	202	414	0.33
<3.5	436	177	374	0.32

The negative predictive value (NPV) is defined as: Number of true negatives/(Number of True negatives + Number of False negatives), where a 'true negative' is the event that the test makes a negative prediction, and a 'false negative' is the event that the test makes a negative prediction, but the subject has a positive result.. Obstructive CAD is defined as ≥70% stenosis in any vessel. *Non-event: <70% stenosis. HsTnI= high sensitivity troponin I

Table S8. Multivariate Cox Regression on incident MI with Age, Sex, Dichotomized Singulex hsTnl and Stepwise Selection on Baseline Biomarkers* and other Baseline Covariates. Full study cohort (N=991).**

Parameter	Parameter Estimate	Standard Error	P-Value	Hazard Ratio	95% CI
Age, years	0.02638	0.00820	0.001	1.03	[1.01, 1.04]
Male sex	0.14521	0.19664	0.46	1.16	[0.79, 1.70]
HsTnl ≥99th percentile, ng/L	0.98506	0.18530	<0.001	2.68	[1.86, 3.85]
Systolic Blood Pressure (mmHg)	-0.01218	0.00402	0.002	0.99	[0.98, 1.00]
NT-ProBNP, pg/mL	0.0000141	0.0000127	0.27	1.00	[1.00, 1.00]
Nitrates	0.50690	0.19422	0.01	1.66	[1.14, 2.43]
MPO, pmol/L	0.0003056	0.0000970	0.002	1.00	[1.00, 1.00]
Diabetes mellitus	0.41814	0.18192	0.02	1.52	[1.06, 2.17]
Coronary Artery Disease	0.48949	0.19240	0.01	1.63	[1.12, 2.38]
Cystatin C, mg/L	0.20456	0.09470	0.03	1.23	[1.02, 1.48]

CI= confidence interval, hsTn= high sensitivity troponin, MPO= myeloperoxidase, NT-proBNP= amino-terminal pro-B type natriuretic peptide

* Baseline Biomarkers include: myeloperoxidase, NT-proBNP= amino-terminal pro-B type natriuretic peptide, Cystatin C.
** Other Covariates include: Heart Rate, Systolic Blood Pressure, Diastolic Blood Pressure, prior MI, Atrial Fibrillation/Flutter, Hypertension, Coronary Artery Disease, Heart Failure, COPD, Diabetes type I/type II, chronic kidney disease, ACE-I/ARB, Beta Blocker, Aldosterone Antagonist, Loop Diuretics, Nitrates, Calcium channel blockers, Sodium, eGFR (MDRD), Glucose, and hemoglobin.

Table S9. Multivariate Cox Regression on incident MI with Age, Sex, Dichotomized Singulex hsTnl and Stepwise Selection on Baseline Biomarkers* and other Baseline Covariates**
Full cohort excluding patients with unstable angina (N=857)

Parameter	Parameter Estimate	Standard Error	P-Value	Hazard Ratio	95% CI
Age, years	0.02705	0.00907	0.003	1.03	[1.01, 1.05]
Male sex	0.30676	0.23064	0.18	1.36	[0.87, 2.14]
HsTnl ≥99th percentile, ng/L	0.88094	0.20710	<0.001	2.41	[1.61, 3.62]
Systolic Blood Pressure (mmHg)	-0.00840	0.00453	0.06	0.99	[0.98, 1.00]
NT-ProBNP, pg/mL	0.0000259	0.0000141	0.07	1.00	[1.00, 1.00]
Nitrates	0.56958	0.23445	0.02	1.77	[1.12, 2.80]
MPO, pmol/L	0.0002943	0.0000997	0.003	1.00	[1.00, 1.00]
Diabetes mellitus	0.73391	0.20108	<0.001	2.08	[1.41, 3.09]
Coronary Artery Disease	0.28271	0.20581	0.17	1.33	[0.89, 1.99]
Cystatin C, mg/L	0.11425	0.11132	0.30	1.12	[0.90, 1.39]

CI= confidence interval, hsTn= high sensitivity troponin, MPO= myeloperoxidase, NT-proBNP= amino-terminal pro-B type natriuretic peptide
* Baseline Biomarkers include: myeloperoxidase, NT-proBNP= amino-terminal pro-B type natriuretic peptide, Cystatin C.
** Other Covariates include: Heart Rate, Systolic Blood Pressure, Diastolic Blood Pressure, Atrial Fibrillation/Flutter, Hypertension, Coronary Artery Disease, Heart Failure, COPD, prior MI, Diabetes type I/type II, chronic kidney disease, ACE-I/ARB, Beta Blocker, Aldosterone Antagonist, Loop Diuretics, Nitrates, Calcium channel blockers, Sodium, eGFR (MDRD), Glucose, and hemoglobin.

Table S10. Multivariate Cox Regression on incident HF with Age, Sex, dichotomized Singulex hsTnl and Stepwise Selection on Baseline Biomarkers* and other Baseline Covariates. Full study cohort (N=991)**

Parameter	Parameter Estimate	Standard Error	P-Value	Hazard Ratio	95% CI
Age, years	0.03669	0.00663	<0.001	1.04	[1.02, 1.05]
Male sex	0.56808	0.15908	<0.001	1.77	[1.29, 2.41]
HsTnl ≥99th percentile	0.47325	0.14462	0.001	1.60	[1.21, 2.13]
NT-ProBNP, pg/mL	0.0000390	7.90921E-6	<0.001	1.00	[1.00, 1.00]
Loop diuretics	0.88738	0.16222	<0.001	2.43	[1.77, 3.34]
Heart Failure	0.38524	0.16032	0.016	1.47	[1.07, 2.01]
Diabetes mellitus	0.44091	0.13877	0.002	1.55	[1.18, 2.04]
COPD	0.63928	0.15331	<0.001	1.90	[1.40, 2.56]
Heart Rate (Beat/min)	0.01323	0.00464	0.004	1.01	[1.00, 1.02]

CI= confidence interval, HF= heart failure, Hgb=hemoglobin, HsTn= high sensitivity troponin, NT-proBNP= amino-terminal pro-B type natriuretic peptide.

* Baseline Biomarkers include: myeloperoxidase, NT-proBNP= amino-terminal pro-B type natriuretic peptide, Cystatin C.
** Other Covariates include: Heart Rate, Systolic Blood Pressure, Diastolic Blood Pressure, Atrial Fibrillation/Flutter, Hypertension, Coronary Artery Disease, Heart Failure, COPD, Diabetes type I/type II, chronic kidney disease, ACE-I/ARB, Beta Blocker, Aldosterone Antagonist, Loop Diuretics, Nitrates, Calcium channel blockers, Sodium, eGFR (MDRD), Glucose, and hemoglobin.

Table S11. Multivariate Cox Regression on incident HF with Age, Sex, dichotomized Singulex hsTnl and Stepwise Selection on Baseline Biomarkers* and other Baseline Covariates.**
Full cohort excluding patients with unstable angina (N=857)

Parameter	Parameter Estimate	Standard Error	P-Value	Hazard Ratio	95% CI
Age, years	0.03872	0.00696	<0.001	1.04	[1.03, 1.05]
Male sex	0.55171	0.16881	0.001	1.74	[1.25, 2.42]
HsTnl ≥99th percentile, ng/L	0.48469	0.15410	0.002	1.62	[1.20, 2.20]
NT-ProBNP, pg/mL	0.0000374	8.87438E-6	<0.001	1.00	[1.00, 1.00]
Loop diuretics	0.78739	0.17362	<0.001	2.20	[1.56, 3.09]
Heart Failure	0.33791	0.17033	0.05	1.40	[1.00, 1.96]
Diabetes mellitus	0.50287	0.14862	<0.001	1.65	[1.24, 2.21]
COPD	0.64522	0.16500	<0.001	1.91	[1.38, 2.63]
Heart Rate (Beat/min)	0.01321	0.00483	0.006	1.01	[1.00, 1.02]

CI= confidence interval, HF= heart failure, Hgb=hemoglobin, HsTn= high sensitivity troponin, NT-proBNP= amino-terminal pro-B type natriuretic peptide.
* Baseline Biomarkers include: myeloperoxidase, NT-proBNP= amino-terminal pro-B type natriuretic peptide, Cystatin C.
** Other Covariates include: Heart Rate, Systolic Blood Pressure, Diastolic Blood Pressure, Atrial Fibrillation/Flutter, Hypertension, Coronary Artery Disease, Heart Failure, COPD, Diabetes type I/type II, chronic kidney disease, ACE-I/ARB, Beta Blocker, Aldosterone Antagonist, Loop Diuretics, Nitrates, Calcium channel blockers, Sodium, eGFR (MDRD), Glucose, and hemoglobin.

Table S12. Multivariate Cox Regression on CV Death with Age, Sex, dichotomized Singulex hsTnl and Stepwise Selection on Baseline Biomarkers and other Baseline Covariates
Full study cohort (N=991)

Parameter	Parameter Estimate	Standard Error	P-Value	Hazard Ratio	95% CI
Age, years	0.05579	0.01106	<0.001	1.06	[1.04, 1.08]
Male sex	0.30526	0.24995	0.2	1.36	[0.83, 2.22]
HsTnl ≥99th percentile, ng/L	0.82854	0.25183	0.001	2.29	[1.40, 3.75]
Systolic Blood Pressure (mmHg)	-0.01138	0.00520	0.03	0.99	[0.98, 1.00]
Loop diuretics	0.65003	0.23666	0.01	1.92	[1.21, 3.05]
Diabetes mellitus	0.51651	0.23036	0.03	1.68	[1.07, 2.63]
COPD	0.50327	0.23663	0.03	1.65	[1.04, 2.63]
Heart Rate (Beat/min)	0.01946	0.00790	0.01	1.02	[1.00 1.04]
Hemoglobin, g/L	-0.19305	0.07358	0.01	0.82	[0.71, 0.95]
Cystatin C, mg/L	0.31438	0.08990	<0.001	1.37	[1.15, 1.63]

CI= confidence interval, COPD=chronic obstructive pulmonary disease, CV= cardiovascular, HsTn= high sensitivity troponin, Hgb=hemoglobin

* Baseline Biomarkers include: myeloperoxidase, NT-proBNP= amino-terminal pro-B type natriuretic peptide, Cystatin C.
** Other Covariates include: Heart Rate, Systolic Blood Pressure, Diastolic Blood Pressure, Atrial Fibrillation/Flutter, Hypertension, Coronary Artery Disease, Heart Failure, COPD, Diabetes type I/type II, chronic kidney disease, ACE-I/ARB, Beta Blocker, Aldosterone Antagonist, Loop Diuretics, Nitrates, Calcium channel blockers, Sodium, eGFR (MDRD), Glucose, and hemoglobin.

Table S13. Multivariate Cox Regression on CV Death with Age, Sex, dichotomized Singulex hsTnl and Stepwise Selection on Baseline Biomarkers* and other Baseline Covariates.**
Full cohort excluding patients with unstable angina (N=857)

Parameter	Parameter Estimate	Standard Error	P-Value	Hazard Ratio	95% CI
Age, years	0.05569	0.01234	<0.001	1.06	[1.03, 1.08]
Male sex	0.43740	0.28272	0.12	1.55	[0.89, 2.70]
HsTnl ≥99th percentile, ng/L	0.85225	0.27476	0.002	2.35	[1.37, 4.02]
Systolic Blood Pressure (mmHg)	-0.01419	0.00612	0.02	0.99	[0.97, 1.00]
Loop diuretics	0.69915	0.26073	0.007	2.01	[1.21, 3.35]
Diabetes mellitus	0.70048	0.25448	0.006	2.02	[1.22, 3.32]
COPD	0.60278	0.25778	0.02	1.83	[1.10, 3.03]
Heart Rate (Beat/min)	0.01633	0.00858	0.06	1.02	[1.00, 1.03]
Hemoglobin, g/L	-0.21858	0.08415	0.009	0.80	[0.68, 0.95]
Cystatin C, mg/L	0.31107	0.10269	0.003	1.37	[1.12, 1.67]

CI= confidence interval, COPD=chronic obstructive pulmonary disease, CV= cardiovascular, HsTn= high sensitivity troponin, Hgb=hemoglobin
* Baseline Biomarkers include: myeloperoxidase, NT-proBNP= amino-terminal pro-B type natriuretic peptide, Cystatin C.
** Other Covariates include: Heart Rate, Systolic Blood Pressure, Diastolic Blood Pressure, Atrial Fibrillation/Flutter, Hypertension, Coronary Artery Disease, Heart Failure, COPD, Diabetes type I/type II, chronic kidney disease, ACE-I/ARB, Beta Blocker, Aldosterone Antagonist, Loop Diuretics, Nitrates, Calcium channel blockers, Sodium, eGFR (MDRD), Glucose, and hemoglobin

Table S14. Multivariate Cox Regression on All Cause Death with Age, Sex, dichotomized Singulex hsTnl and Stepwise Selection on Baseline Biomarkers* and other Baseline Covariates**
Full study cohort (N=991)

Parameter	Parameter Estimate	Standard Error	P-Value	Hazard Ratio	95% CI
Age, years	0.05955	0.00938	<0.001	1.06	[1.04, 1.08]
Male sex	0.43039	0.22152	0.05	1.54	[1.00, 2.37]
HsTnl ≥99th percentile, ng/L	0.61168	0.21022	0.004	1.84	[1.22, 2.78]
Systolic Blood Pressure (mmHg)	-0.01182	0.00441	0.01	0.99	[0.98, 1.00]
NT-ProBNP, pg/mL	0.0000334	9.2877E-6	<0.001	1.00	[1.00, 1.00]
Hypertension	0.57284	0.27794	0.04	1.77	[1.03, 3.06]
Diabetes mellitus	0.54654	0.19574	0.01	1.73	[1.18, 2.54]
COPD	0.70130	0.20032	<0.001	2.02	[1.36, 2.99]
Heart Rate (Beat/min)	0.01705	0.00692	0.01	1.02	[1.00, 1.03]
Hemoglobin, g/L	-0.23833	0.05744	<0.001	0.79	[0.70, 0.88]

CI= confidence interval, COPD= chronic obstructive pulmonary disease, Hgb=hemoglobin, HsTn= high sensitivity troponin, NT-proBNP= amino-terminal pro-B type natriuretic peptide.

* Baseline Biomarkers include: myeloperoxidase, NT-proBNP= amino-terminal pro-B type natriuretic peptide, Cystatin C.
** Other Covariates include: Heart Rate, Systolic Blood Pressure, Diastolic Blood Pressure, Atrial Fibrillation/Flutter, Hypertension, Coronary Artery Disease, Heart Failure, COPD, Diabetes type I/type II, chronic kidney disease, ACE-I/ARB, Beta Blocker, Aldosterone Antagonist, Loop Diuretics, Nitrates, Calcium channel blockers, Sodium, eGFR (MDRD), Glucose, and hemoglobin.

Table S15. Multivariate Cox Regression on All Cause Death with Age, Sex, dichotomized Singulex hsTnl and Stepwise Selection on Baseline Biomarkers* and other Baseline Covariates.**
Full cohort excluding patients with unstable angina (N=857)

Parameter	Parameter Estimate	Standard Error	P-Value	Hazard Ratio	95% CI
Age, years	0.06161	0.01032	<0.001	1.06	[1.04, 1.09]
Male sex	0.61197	0.24485	0.01	1.84	[1.14, 2.98]
HsTnl ≥99th percentile, ng/L	0.61727	0.22514	0.01	1.85	[1.19, 2.88]
Systolic Blood Pressure (mmHg)	-0.01593	0.00516	0.002	0.98	[0.97, 0.99]
NT-ProBNP, pg/mL	0.0000370	0.0000101	<0.001	1.00	[1.00, 1.00]
Hypertension	0.57350	0.29622	0.05	1.77	[0.99, 3.17]
Diabetes mellitus	0.79824	0.21360	<0.001	2.22	[1.46, 3.38]
COPD	0.84285	0.21578	<0.001	2.32	[1.52, 3.55]
Heart Rate (Beat/min)	0.01333	0.00733	0.07	1.01	[1.00, 1.03]
Hemoglobin, g/L	-0.28372	0.06413	<0.001	0.75	[0.66, 0.85]

CI= confidence interval, COPD= chronic obstructive pulmonary disease, Hgb=hemoglobin, HsTn= high sensitivity troponin, NT-proBNP= amino-terminal pro-B type natriuretic peptide.
* Baseline Biomarkers include: myeloperoxidase, NT-proBNP= amino-terminal pro-B type natriuretic peptide, Cystatin C.
** Other Covariates include: Heart Rate, Systolic Blood Pressure, Diastolic Blood Pressure, Atrial Fibrillation/Flutter, Hypertension, Coronary Artery Disease, Heart Failure, COPD, Diabetes type I/type II, chronic kidney disease, ACE-I/ARB, Beta Blocker, Aldosterone Antagonist, Loop Diuretics, Nitrates, Calcium channel blockers, Sodium, eGFR (MDRD), Glucose, and hemoglobin

Table S16. Multivariate Cox Regression on Composite Endpoint of MI, HF and All Cause Death with Age, Sex, dichotomized Singulex hsTnl and Stepwise Selection on Baseline Biomarkers and other Baseline Covariates
Full study cohort (N=991)

Parameter	Parameter Estimate	Standard Error	P-Value	Hazard Ratio	95% CI
Age, years	0.03766	0.00601	<0.001	1.04	[1.03, 1.05]
Male sex	0.51648	0.14057	<0.001	1.68	[1.27, 2.21]
HsTnl ≥99th percentile, ng/L	0.48335	0.12706	<0.001	1.62	[1.26, 2.08]
Systolic Blood Pressure (mmHg)	-0.00867	0.00281	0.002	0.99	[0.99, 1.00]
NT-ProBNP, pg/mL	0.0000415	6.8046E-6	<0.001	1.00	[1.00, 1.00]
Loop diuretics	0.77036	0.12992	<0.001	2.16	[1.68, 2.79]
Diabetes mellitus	0.56125	0.12268	<0.001	1.75	[1.38, 2.23]
COPD	0.60748	0.13742	<0.001	1.84	[1.40, 2.40]
Heart Rate (Beat/min)	0.01258	0.00425	0.003	1.01	[1.00, 1.02]
Hemoglobin, g/L	-0.08915	0.03863	0.02	0.92	[0.85, 0.99]

CI= confidence interval, COPD= chronic obstructive pulmonary disease, HF= heart failure, Hgb=hemoglobin, HsTn= high sensitivity troponin, MI= myocardial infarction, NT-proBNP= amino-terminal pro-B type natriuretic peptide.

* Baseline Biomarkers include: myeloperoxidase, NT-proBNP= amino-terminal pro-B type natriuretic peptide, Cystatin C.
** Other Covariates include: Heart Rate, Systolic Blood Pressure, Diastolic Blood Pressure, Atrial Fibrillation/Flutter, Hypertension, Coronary Artery Disease, Heart Failure, COPD, Diabetes type I/type II, chronic kidney disease, ACE-I/ARB, Beta Blocker, Aldosterone Antagonist, Loop Diuretics, Nitrates, Calcium channel blockers, Sodium, eGFR (MDRD), Glucose, and hemoglobin.

Table S17. Multivariate Cox Regression on Composite Endpoint of MI, HF and All Cause Death with Age, Sex, dichotomized Singulex hsTnl and Stepwise Selection on Baseline Biomarkers* and other Baseline Covariates.**
Full cohort excluding patients with unstable angina (N=857)

Parameter	Parameter Estimate	Standard Error	P-Value	Hazard Ratio	95% CI
Age, years	0.03946	0.00640	<0.001	1.04	[1.03, 1.05]
Male sex	0.50664	0.15237	<0.001	1.66	[1.23, 2.24]
HsTnl ≥99th percentile, ng/L	0.45629	0.13832	0.001	1.58	[1.20, 2.07]
Systolic Blood Pressure (mmHg)	-0.00855	0.00313	0.01	0.99	[0.99, 1.00]
NT-ProBNP, pg/mL	0.0000429	7.54771E-6	<0.001	1.00	[1.00, 1.00]
Loop diuretics	0.73766	0.14090	<0.001	2.09	[1.59, 2.76]
Diabetes mellitus	0.70205	0.13326	<0.001	2.02	[1.55, 2.62]
COPD	0.66864	0.14861	<0.001	1.95	[1.46, 2.61]
Heart Rate (Beat/min)	0.01250	0.00443	0.005	1.01	[1.00, 1.02]
Hemoglobin, g/L	-0.06452	0.04154	0.12	0.94	[0.86, 1.02]

CI= confidence interval, COPD= chronic obstructive pulmonary disease, HF= heart failure, Hgb=hemoglobin, HsTn= high sensitivity troponin, MI= myocardial infarction, NT-proBNP= amino-terminal pro-B type natriuretic peptide.

* Baseline Biomarkers include: myeloperoxidase, NT-proBNP= amino-terminal pro-B type natriuretic peptide, Cystatin C.
** Other Covariates include: Heart Rate, Systolic Blood Pressure, Diastolic Blood Pressure, Atrial Fibrillation/Flutter, Hypertension, Coronary Artery Disease, Heart Failure, COPD, Diabetes type I/type II, chronic kidney disease, ACE-I/ARB, Beta Blocker, Aldosterone Antagonist, Loop Diuretics, Nitrates, Calcium channel blockers, Sodium, eGFR (MDRD), Glucose, and hemoglobin.

Table S18. Multivariate Cox Regression on Composite Endpoint of MI and CV Death with Age, Sex, dichotomized Singulex hsTnl and Stepwise Selection on Baseline Biomarkers* and other Baseline Covariates.**
Full study cohort (N=991)

Parameter	Parameter Estimate	Standard Error	P-Value	Hazard Ratio	95% CI
Age, years	0.03609	0.00748	<0.001	1.04	[1.02, 1.05]
Male sex	0.31861	0.17967	0.08	1.38	[0.97, 1.96]
HsTnl ≥99th percentile	0.79862	0.16666	<0.001	2.22	[1.60, 3.08]
NT-ProBNP, pg/mL	0.0000189	0.0000105	0.07	1.00	[1.00, 1.00]
Nitrates	0.56157	0.17042	0.001	1.75	[1.26, 2.45]
MPO, pmol/L	0.0002353	0.0000985	0.02	1.00	[1.00, 1.00]
Diabetes mellitus	0.58768	0.15892	<0.001	1.80	[1.32, 2.46]
COPD	0.59110	0.17202	<0.001	1.81	[1.29, 2.53]
Heart Rate (Beat/min)	0.01415	0.00562	0.01	1.01	[1.00, 1.03]
Hemoglobin, g/L	-0.16696	0.05114	0.001	0.85	[0.77, 0.94]
Cystatin C, mg/L	0.15804	0.08546	0.06	1.17	[0.99, 1.39]

CI= confidence interval, COPD= chronic obstructive pulmonary disease, CV= cardiovascular, Hgb=hemoglobin, HsTn= high sensitivity troponin, MI= myocardial infarction NT-proBNP= amino-terminal pro-B type natriuretic peptide.
* Baseline Biomarkers include: myeloperoxidase, NT-proBNP= amino-terminal pro-B type natriuretic peptide, Cystatin C.
** Other Covariates include: Heart Rate, Systolic Blood Pressure, Diastolic Blood Pressure, Atrial Fibrillation/Flutter, Hypertension, Coronary Artery Disease, Heart Failure, COPD, Diabetes type I/type II, chronic kidney disease, ACE-I/ARB, Beta Blocker, Aldosterone Antagonist, Loop Diuretics, Nitrates, Calcium channel blockers, Sodium, eGFR (MDRD), Glucose, and hemoglobin.

Table S19. Multivariate Cox Regression on Composite Endpoint of MI and CV Death with Age, Sex, dichotomized Singulex hsTnl and Stepwise Selection on Baseline Biomarkers* and other Baseline Covariates.**
Full cohort excluding patients with unstable angina (N=857)

Parameter	Parameter Estimate	Standard Error	P-Value	Hazard Ratio	95% CI
Age, years	0.03938	0.00830	<0.001	1.04	[1.02, 1.06]
Male sex	0.36307	0.20155	0.07	1.44	[0.97, 2.13]
HsTnl ≥99th percentile, ng/L	0.72370	0.18640	<0.001	2.06	[1.43, 2.97]
NT-ProBNP, pg/mL	0.0000302	0.0000106	0.005	1.00	[1.00, 1.00]
Nitrates	0.65744	0.20629	0.001	1.93	[1.29, 2.89]
MPO, pmol/L	0.0002479	0.0000998	0.01	1.00	[1.00, 1.00]
Diabetes mellitus	0.84880	0.17642	<0.001	2.34	[1.65, 3.30]
COPD	0.53003	0.19288	0.006	1.70	[1.16, 2.48]
Heart Rate (Beat/min)	0.01442	0.00604	0.02	1.02	[1.00, 1.03]
Hemoglobin, g/L	-0.12124	0.05652	0.03	0.89	[0.79, 0.99]
Cystatin C, mg/L	0.15771	0.09595	0.10	1.17	[0.97, 1.41]

CI= confidence interval, COPD= chronic obstructive pulmonary disease, CV= cardiovascular, Hgb=hemoglobin, HsTn= high sensitivity troponin, MI= myocardial infarction NT-proBNP= amino-terminal pro-B type natriuretic peptide.
* Baseline Biomarkers include: myeloperoxidase, NT-proBNP= amino-terminal pro-B type natriuretic peptide, Cystatin C.
** Other Covariates include: Heart Rate, Systolic Blood Pressure, Diastolic Blood Pressure, Atrial Fibrillation/Flutter, Hypertension, Coronary Artery Disease, Heart Failure, COPD, Diabetes type I/type II, chronic kidney disease, ACE-I/ARB, Beta Blocker, Aldosterone Antagonist, Loop Diuretics, Nitrates, Calcium channel blockers, Sodium, eGFR (MDRD), Glucose, and hemoglobin.

Table S20. Multivariate Cox Regression on Composite Endpoint of MI and All Cause Death with Age, Sex, dichotomized Singulex hsTnl and Stepwise Selection on Baseline Biomarkers* and other Baseline Covariates.**
Full study cohort (N=991)

Parameter	Parameter Estimate	Standard Error	P-Value	Hazard Ratio	95% CI
Age, years	0.04086	0.00735	<0.001	1.04	[1.03, 1.06]
Male sex	0.32068	0.17234	0.06	1.38	[0.98, 1.93]
HsTnl ≥99th percentile, ng/L	0.75474	0.15821	<0.001	2.13	[1.56, 2.90]
Systolic Blood Pressure (mmHg)	-0.01051	0.00354	0.003	0.99	[0.98, 1.00]
NT-ProBNP, pg/mL	0.0000186	9.97975E-6	0.06	1.00	[1.00, 1.00]
Nitrates	0.49934	0.16412	0.002	1.65	[1.19, 2.27]
MPO, pmol/L	0.0002380	0.0000935	0.01	1.00	[1.00, 1.00]
Diabetes mellitus	0.53927	0.15348	<0.001	1.72	[1.27, 2.32]
COPD	0.65013	0.16100	<0.001	1.92	[1.40, 2.63]
Heart Rate (Beat/min)	0.01220	0.00542	0.02	1.01	[1.00, 1.02]
Hemoglobin, g/L	-0.15133	0.04934	0.002	0.86	[0.78, 0.95]
Cystatin C, mg/L	0.18724	0.08211	0.02	1.21	[1.03, 1.42]

CI= confidence interval, COPD= chronic obstructive pulmonary disease, Hgb=hemoglobin, HsTn= high sensitivity troponin, MI= myocardial infarction, MPO=myeloperoxidase, NT-proBNP= amino-terminal pro-B type natriuretic peptide.
* Baseline Biomarkers include: myeloperoxidase, NT-proBNP= amino-terminal pro-B type natriuretic peptide, Cystatin C.
** Other Covariates include: Heart Rate, Systolic Blood Pressure, Diastolic Blood Pressure, Atrial Fibrillation/Flutter, Hypertension, Coronary Artery Disease, Heart Failure, COPD, Diabetes type I/type II, chronic kidney disease, ACE-I/ARB, Beta Blocker, Aldosterone Antagonist, Loop Diuretics, Nitrates, Calcium channel blockers, Sodium, eGFR (MDRD), Glucose, and hemoglobin.

Table S21. Multivariate Cox Regression on Composite Endpoint of MI and All Cause Death with Age, Sex, dichotomized Singulex hsTnl and Stepwise Selection on Baseline Biomarkers* and other Baseline Covariates**
Full cohort excluding patients with unstable angina (N=857)

Parameter	Parameter Estimate	Standard Error	P-Value	Hazard Ratio	95% CI
Age, years	0.04130	0.00806	<0.001	1.04	[1.03, 1.06]
Male sex	0.33994	0.19198	0.08	1.41	[0.96, 2.05]
HsTnl ≥99th percentile, ng/L	0.68177	0.17603	<0.001	1.98	[1.40, 2.79]
Systolic Blood Pressure (mmHg)	-0.00844	0.00398	0.03	0.99	[0.98, 1.00]
NT-ProBNP, pg/mL	0.0000275	0.0000103	0.01	1.00	[1.00, 1.00]
Nitrates	0.58695	0.19730	0.003	1.80	[1.22, 2.65]
MPO, pmol/L	0.0002599	0.0000924	0.005	1.00	[1.00, 1.00]
Diabetes mellitus	0.79670	0.16966	<0.001	2.22	[1.59, 3.09]
COPD	0.63817	0.17849	0.0003	1.89	[1.33, 2.69]
Heart Rate (Beat/min)	0.01309	0.00580	0.02	1.01	[1.00, 1.03]
Hemoglobin, g/L	-0.11588	0.05411	0.03	0.89	[0.80, 0.99]
Cystatin C, mg/L	0.16508	0.09310	0.08	1.18	[0.98, 1.42]

CI= confidence interval, COPD= chronic obstructive pulmonary disease, Hgb=hemoglobin, HsTn= high sensitivity troponin, MI= myocardial infarction, MPO=myeloperoxidase, NT-proBNP= amino-terminal pro-B type natriuretic peptide.
* Baseline Biomarkers include: myeloperoxidase, NT-proBNP= amino-terminal pro-B type natriuretic peptide, Cystatin C.
** Other Covariates include: Heart Rate, Systolic Blood Pressure, Diastolic Blood Pressure, Atrial Fibrillation/Flutter, Hypertension, Coronary Artery Disease, Heart Failure, COPD, Diabetes type I/type II, chronic kidney disease, ACE-I/ARB, Beta Blocker, Aldosterone Antagonist, Loop Diuretics, Nitrates, Calcium channel blockers, Sodium, eGFR (MDRD), Glucose, and hemoglobin.

Table S22. Multivariate Cox Proportional Hazards Regression on incident MI, with age, sex, dichotomized Singulex hsTnl and other covariates*
For Male (N = 713)

Parameter	Parameter Estimate	Standard Error	p-value	Hazard Ratio	95% CI
Age, years	0.03083	0.01038	0.003	1.03	[1.01, 1.05]
HsTnl ≥99th percentile (ng/L)	0.95677	0.22252	<0.001	2.60	[1.68, 4.03]
Systolic Blood Pressure (mmHg)	-0.01211	0.00517	0.02	0.99	[0.98, 1.00]
Diabetes mellitus	0.50384	0.21662	0.02	1.66	[1.08, 2.53]
Nitrates	0.62803	0.22123	0.005	1.87	[1.21, 2.89]
Hemoglobin, g/L	-0.14478	0.06655	0.03	0.87	[0.76, 0.99]
MPO (pmol/L)	0.0002409	0.0001090	0.03	1.00	[1.00, 1.00]
Cystatin C (mg/L)	0.29044	0.07925	<0.001	1.34	[1.14, 1.56]

CI= confidence interval, hsTn= high sensitivity troponin, Hgb=hemoglobin, MI= myocardial infarction, MPO=myeloperoxidase.

***Other Covariates include**: Heart Rate, Systolic Blood Pressure, Diastolic Blood Pressure, Atrial Fibrillation/Flutter, Hypertension, Coronary Artery Disease, Heart Failure, COPD, Diabetes type I/type II, CKD, ACE-I/ARB, Beta Blocker, Aldosterone Antagonist, Loop Diuretics, Nitrates, CCB, Sodium, GFR (MDRD), Glucose, and HGB.
Stepwise selection: levels for entry into and staying in the model are both 0.1.

Table S23. Multivariate Cox Proportional Hazards Regression on incident MI, with age, sex, dichotomized Singulex hsTnl and other covariates* For Female (N = 278)

Parameter	Parameter Estimate	Standard Error	p-value	Hazard Ratio	95% CI
Age, years	0.00222	0.01801	0.90	1.00	[0.97, 1.04]
HsTnl ≥99th percentile (ng/L)	0.95927	0.38309	0.01	2.61	[1.23, 5.53]
Hypertension	1.44643	0.75520	0.06	4.25	[0.97, 18.66]
COPD	1.01969	0.39375	0.01	2.77	[1.28, 6.00]
Diabetes mellitus	0.79900	0.40956	0.05	2.22	[1.00, 4.96]
Nitrates	1.13389	0.40922	0.01	3.11	[1.39, 6.93]
Glucose (mg/dL)	0.00778	0.00356	0.03	1.01	[1.00, 1.01]

CI= confidence interval, COPD= chronic obstructive pulmonary disease, hsTn= high sensitivity troponin, MI= myocardial infarction.

***Other Covariates include**: Heart Rate, Systolic Blood Pressure, Diastolic Blood Pressure, Atrial Fibrillation/Flutter, Hypertension, Coronary Artery Disease, Heart Failure, COPD, Diabetes type I/type II, CKD, ACE-I/ARB, Beta Blocker, Aldosterone Antagonist, Loop Diuretics, Nitrates, CCB, Sodium, GFR (MDRD), Glucose, and HGB.
Stepwise selection: levels for entry into and staying in the model are both 0.1.

Table S24. Multivariate Cox Proportional Hazards Regression on incident CHF, with age, sex, dichotomized Singulex hsTnl and other covariates* forced into the model
For Male (N = 713)

Parameter	Parameter Estimate	Standard Error	p-value	Hazard Ratio	95% CI
Age	0.03362	0.00785	<0.001	1.03	[1.02, 1.05]
HsTnl ≥99th percentile (ng/L)	0.53737	0.16147	<0.001	1.71	[1.25, 2.35]
Heart Rate (Beat/min)	0.01358	0.00511	0.01	1.01	[1.00, 1.02]
Atrial Fibrillation/Flutter	0.67202	0.16448	<0.001	1.96	[1.42, 2.70]
COPD	0.69097	0.17745	<0.001	2.00	[1.41, 2.83]
Loop diuretics	1.12166	0.16760	<0.001	3.07	[2.21, 4.26]
NT-ProBNP (pg/mL)	0.0000581	9.16058E-6	<0.001	1.00	[1.00, 1.00]

CI= confidence interval, CHF= congestive heart failure, COPD= chronic obstructive pulmonary disease, hsTn= high sensitivity troponin, NT-proBNP= amino-terminal pro-B type natriuretic peptide.

***Other Covariates include**: Heart Rate, Systolic Blood Pressure, Diastolic Blood Pressure, Atrial Fibrillation/Flutter, Hypertension, Coronary Artery Disease, Heart Failure, COPD, Diabetes type I/type II, CKD, ACE-I/ARB, Beta Blocker, Aldosterone Antagonist, Loop Diuretics, Nitrates, CCB, Sodium, GFR (MDRD), Glucose, and HGB.
Stepwise selection: levels for entry into and staying in the model are both 0.1.

Table S25. Multivariate Cox Proportional Hazards Regression on incident CHF, with age, sex, dichotomized Singulex hsTnl and other covariates*
For Female (N = 278)

Parameter	Parameter Estimate	Standard Error	p-value	Hazard Ratio	95% CI
Age, years	0.02036	0.01256	0.11	1.02	[1.00, 1.05]
HsTnl ≥99th percentile (ng/L)	0.30941	0.32337	0.34	1.36	[0.72, 2.57]
Heart Failure	1.62016	0.32053	<0.001	5.05	[2.70, 9.47]
Diabetes mellitus	1.13633	0.30075	<0.001	3.12	[1.73, 5.62]
CKD	0.86948	0.39454	0.03	2.39	[1.10, 5.17]
Sodium (mmol/L)	-0.16064	0.04022	<0.001	0.85	[0.79, 0.92]

***Other Covariates include:** Heart Rate, Systolic Blood Pressure, Diastolic Blood Pressure, Atrial Fibrillation/Flutter, Hypertension, Coronary Artery Disease, Heart Failure, COPD, Diabetes type I/type II, CKD, ACE-I/ARB, Beta Blocker, Aldosterone Antagonist, Loop Diuretics, Nitrates, CCB, Sodium, GFR (MDRD), Glucose, and HGB.
Stepwise selection: levels for entry into and staying in the model are both 0.1.

Table S26. Multivariate Cox Proportional Hazards Regression on CV death, with age, sex, dichotomized Singulex hsTnl and other covariates*
For Male (N = 713)

Parameter	Parameter Estimate	Standard Error	p-value	Hazard Ratio	95% CI
Age, years	0.04255	0.01270	<0.001	1.04	[1.02, 1.07]
HsTnl ≥99th percentile (ng/L)	0.82429	0.29010	0.004	2.28	[1.29, 4.03]
Heart Rate (Beat/min)	0.01759	0.00932	0.06	1.02	[1.00, 1.04]
Diastolic Blood Pressure (mmHg)	-0.01669	0.01142	0.14	0.98	[0.96, 1.01]
Diabetes mellitus	0.53499	0.26395	0.04	1.71	[1.02, 2.86]
Loop diuretics	1.01605	0.26600	<0.001	2.76	[1.64, 4.65]
Hemoglobin, g/L	-0.23161	0.08187	0.005	0.79	[0.68, 0.93]
Cystatin C (mg/L)	0.30633	0.09799	0.002	1.36	[1.12, 1.65]

CI= confidence interval, CV= cardiovascular, Hgb=hemoglobin, hsTn= high sensitivity troponin.

***Other Covariates include**: Heart Rate, Systolic Blood Pressure, Diastolic Blood Pressure, Atrial Fibrillation/Flutter, Hypertension, Coronary Artery Disease, Heart Failure, COPD, Diabetes type I/type II, CKD, ACE-I/ARB, Beta Blocker, Aldosterone Antagonist, Loop Diuretics, Nitrates, CCB, Sodium, GFR (MDRD), Glucose, and HGB.
Stepwise selection: levels for entry into and staying in the model are both 0.1.

Table S27. Multivariate Cox Proportional Hazards Regression on CV death, with age, sex, dichotomized Singulex hsTnl and other covariates*
For Female (N = 278)

Parameter	Parameter Estimate	Standard Error	p-value	Hazard Ratio	95% CI
Age, years	0.07451	0.01907	<0.001	1.08	[1.04, 1.12]
HsTnl ≥99th percentile (ng/L)	0.79162	0.43329	0.07	2.21	[0.94, 5.16]
Heart Rate (Beat/min)	0.03395	0.01402	0.02	1.03	[1.01, 1.06]
Beta blocker	1.06697	0.54144	0.05	2.91	[1.01, 8.40]
Cystatin C (mg/L)	0.47908	0.16958	0.005	1.61	[1.16, 2.25]

CI= confidence interval, CV= cardiovascular, hsTn= high sensitivity troponin.

***Other Covariates include**: Heart Rate, Systolic Blood Pressure, Diastolic Blood Pressure, Atrial Fibrillation/Flutter, Hypertension, Coronary Artery Disease, Heart Failure, COPD, Diabetes type I/type II, CKD, ACE-I/ARB, Beta Blocker, Aldosterone Antagonist, Loop Diuretics, Nitrates, CCB, Sodium, GFR (MDRD), Glucose, and HGB.
Stepwise selection: levels for entry into and staying in the model are both 0.1.

Table S28. Multivariate Cox Proportional Hazards Regression on all-cause death, with age, sex, dichotomized Singulex hsTnl and other covariates*
For Male (N = 713)

Parameter	Parameter Estimate	Standard Error	p-value	Hazard Ratio	95% CI
Age, years	0.05349	0.01110	<0.001	1.05	[1.03, 1.08]
HsTnl ≥99th percentile (ng/L)	0.39117	0.23905	0.10	1.48	[0.93, 2.36]
Heart Rate (Beat/min)	0.01612	0.00786	0.04	1.02	[1.00, 1.03]
Diastolic Blood Pressure (mmHg)	-0.01236	0.00974	0.20	0.99	[0.97, 1.01]
Diabetes mellitus	0.49708	0.22217	0.03	1.64	[1.06, 2.54]
Loop diuretics	0.70036	0.22395	0.002	2.01	[1.30, 3.12]
Hemoglobin, g/L	-0.26702	0.06522	<0.001	0.77	[0.67, 0.87]
NT-ProBNP (pg/mL)	0.0000387	0.0000103	<0.001	1.00	[1.00, 1.00]

CI= confidence interval, Hgb=hemoglobin, hsTn= high sensitivity troponin, NT-proBNP= amino-terminal pro-B type natriuretic peptide.

***Other Covariates include:** Heart Rate, Systolic Blood Pressure, Diastolic Blood Pressure, Atrial Fibrillation/Flutter, Hypertension, Coronary Artery Disease, Heart Failure, COPD, Diabetes type I/type II, CKD, ACE-I/ARB, Beta Blocker, Aldosterone Antagonist, Loop Diuretics, Nitrates, CCB, Sodium, GFR (MDRD), Glucose, and HGB.
Stepwise selection: levels for entry into and staying in the model are both 0.1.

Table S29. Multivariate Cox Proportional Hazards Regression on all-cause death, with age, sex, dichotomized Singulex hsTnl and other covariates*
For Female (N = 278)

Parameter	Parameter Estimate	Standard Error	p-value	Hazard Ratio	95% CI
Age, years	0.05828	0.02162	0.01	1.06	[1.02, 1.11]
HsTnl ≥99th percentile (ng/L)	1.76284	0.44445	<0.001	5.83	[2.44, 13.93]
COPD	1.42258	0.40388	<0.001	4.15	[1.88, 9.15]
Beta blocker	1.15640	0.54554	0.03	3.18	[1.09, 9.26]
Glucose (mg/dL)	0.01005	0.00386	0.01	1.01	[1.00, 1.02]

CI= confidence interval, COPD=chronic obstructive pulmonary disease, hsTn= high sensitivity troponin.

***Other Covariates include:** Heart Rate, Systolic Blood Pressure, Diastolic Blood Pressure, Atrial Fibrillation/Flutter, Hypertension, Coronary Artery Disease, Heart Failure, COPD, Diabetes type I/type II, CKD, ACE-I/ARB, Beta Blocker, Aldosterone Antagonist, Loop Diuretics, Nitrates, CCB, Sodium, GFR (MDRD), Glucose, and HGB.
Stepwise selection: levels for entry into and staying in the model are both 0.1.

Table S30. Multivariate Cox Proportional Hazards Regression on incident MI, with age, sex, dichotomized Singulex hsTnl and other covariates*
Patients with obstructive CAD only (N=619)

Parameter	Parameter Estimate	Standard Error	P-Value	Hazard Ratio	95% CI
Age, years	0.04751	0.01138	<0.001	1.05	[1.03, 1.07]
Male sex	0.07181	0.26171	0.78	1.07	[0.64, 1.79]
HsTnl ≥99th percentile, (ng/L)	0.62400	0.24158	0.01	1.87	[1.16, 3.00]
Systolic Blood Pressure (mmHg)	-0.01155	0.00536	0.03	0.99	[0.98, 1.00]
Coronary Artery Disease	0.44404	0.26942	0.10	1.56	[0.92, 2.64]
COPD	0.52674	0.25953	0.04	1.69	[1.02, 2.82]
Diabetes mellitus	0.37893	0.22841	0.10	1.46	[0.93, 2.29]
Nitrates	0.63144	0.23665	0.01	1.88	[1.18, 2.99]
MPO (pmol/L)	0.0002727	0.0001270	0.03	1.00	[1.00, 1.00]
NT-ProBNP (pg/mL)	0.0000377	0.0000153	0.01	1.00	[1.00, 1.00]
Cystatin C (mg/L)	0.23064	0.13211	0.08	1.26	[0.97, 1.63]

CI= confidence interval, COPD= chronic obstructive pulmonary disease, HsTn= high sensitivity troponin, MI= myocardial infarction, MPO=myeloperoxidase, NT-proBNP= amino-terminal pro-B type natriuretic peptide.

***Other Covariates include**: Heart Rate, Systolic Blood Pressure, Diastolic Blood Pressure, Atrial Fibrillation/Flutter, Hypertension, Coronary Artery Disease, Heart Failure, COPD, Diabetes type I/type II, CKD, ACE-I/ARB, Beta Blocker, Aldosterone Antagonist, Loop Diuretics, Nitrates, CCB, Sodium, GFR (MDRD), Glucose, and HGB.
Stepwise selection: levels for entry into and staying in the model are both 0.1.

Table S31. Multivariate Cox Proportional Hazards Regression on CV Mortality, with age, sex, dichotomized Singulex hsTnl and other covariates*
Patients with obstructive CAD only (N=619)

Parameter	Parameter Estimate	Standard Error	P-Value	Hazard Ratio	95% CI
Age, years	0.05349	0.01271	<0.001	1.05	[1.03, 1.08]
Male sex	-0.13892	0.29444	0.64	0.87	[0.49, 1.55]
HsTnl ≥99th percentile, (ng/L)	1.00752	0.31334	0.001	2.74	[1.48, 5.06]
Heart Rate (Beat/min)	0.01646	0.00899	0.07	1.02	[1.00, 1.03]
Systolic Blood Pressure (mmHg)	-0.01458	0.00598	0.02	0.99	[0.97, 1.00]
COPD	0.55026	0.28536	0.05	1.73	[0.99, 3.03]
Loop diuretics	0.66155	0.26539	0.01	1.94	[1.15, 3.26]
CCB	0.56964	0.26125	0.03	1.77	[1.06, 2.95]
Cystatin C (mg/L)	0.39872	0.08805	<0.001	1.49	[1.25, 1.77]

CI= confidence interval, CCB= calcium channel blockers, hsTn= high sensitivity troponin, Hgb=hemoglobin, MI= myocardial infarction, MPO=myeloperoxidase,

***Other Covariates include**: Heart Rate, Systolic Blood Pressure, Diastolic Blood Pressure, Atrial Fibrillation/Flutter, Hypertension, Coronary Artery Disease, Heart Failure, COPD, Diabetes type I/type II, CKD, ACE-I/ARB, Beta Blocker, Aldosterone Antagonist, Loop Diuretics, Nitrates, CCB, Sodium, GFR (MDRD), Glucose, and HGB.
Stepwise selection: levels for entry into and staying in the model are both 0.1.

Table S32. Multivariate Cox Proportional Hazards Regression on incident CHF, with age, sex, dichotomized Singulex hsTnl and other covariates*
Patients with obstructive CAD only (N=619)

Parameter	Parameter Estimate	Standard Error	P-Value	Hazard Ratio	95% CI
Age, years	0.05146	0.01007	<0.001	1.05	[1.03, 1.07]
Male sex	0.27257	0.24558	0.27	1.31	[0.81, 2.13]
HsTnl ≥99th percentile (ng/L)	0.16095	0.21890	0.46	1.17	[0.76, 1.80]
Atrial Fibrillation/Flutter	0.55022	0.21357	0.01	1.73	[1.14, 2.63]
Heart Failure	0.43970	0.23555	0.06	1.55	[0.98, 2.46]
COPD	0.82314	0.22080	<0.001	2.28	[1.48, 3.51]
Diabetes mellitus	0.64951	0.19733	<0.001	1.91	[1.30, 2.82]
Loop diuretics	0.81967	0.23854	<0.001	2.27	[1.42, 3.62]
NT-ProBNP (pg/mL)	0.0000351	0.0000122	0.004	1.00	[1.00, 1.00]

CI= confidence interval, CHF= congestive heart failure, COPD= chronic obstructive pulmonary disease, HsTn= high sensitivity troponin, MPO=myeloperoxidase, NT-proBNP= amino-terminal pro-B type natriuretic peptide.

***Other Covariates include**: Heart Rate, Systolic Blood Pressure, Diastolic Blood Pressure, Atrial Fibrillation/Flutter, Hypertension, Coronary Artery Disease, Heart Failure, COPD, Diabetes type I/type II, CKD, ACE-I/ARB, Beta Blocker, Aldosterone Antagonist, Loop Diuretics, Nitrates, CCB, Sodium, GFR (MDRD), Glucose, and HGB.
Stepwise selection: levels for entry into and staying in the model are both 0.1.

Table S33. Multivariate Cox Proportional Hazards Regression on All-Cause Mortality, with age, sex, dichotomized Singulex hsTnl and other covariates*
Patients with obstructive CAD only (N=619)

Parameter	Parameter Estimate	Standard Error	P-Value	Hazard Ratio	95% CI
Age, years	0.06097	0.01073	<0.001	1.06	[1.04, 1.09]
Male sex	0.12664	0.26054	0.63	1.14	[0.68, 1.89]
HsTnl ≥99th percentile (ng/L)	0.72151	0.25124	0.004	2.06	[1.26, 3.37]
Heart Rate (Beat/min)	0.01830	0.00820	0.03	1.02	[1.00, 1.03]
Systolic Blood Pressure (mmHg)	-0.01290	0.00495	0.01	0.99	[0.98, 1.00]
COPD	0.68097	0.24388	0.01	1.98	[1.23, 3.19]
Diabetes mellitus	0.42781	0.22177	0.05	1.53	[0.99, 2.37]
Nitrates	0.50634	0.22917	0.03	1.66	[1.06, 2.60]
Hemoglobin, g/L	-0.17093	0.06937	0.01	0.84	[0.74, 0.97]
NT-ProBNP (pg/mL)	0.0000390	0.0000103	<0.001	1.00	[1.00, 1.00]

CI= confidence interval, HF= heart failure, hsTn= high sensitivity troponin, NT-proBNP= amino-terminal pro-B type natriuretic peptide.

***Other Covariates include**: Heart Rate, Systolic Blood Pressure, Diastolic Blood Pressure, Atrial Fibrillation/Flutter, Hypertension, Coronary Artery Disease, Heart Failure, COPD, Diabetes type I/type II, CKD, ACE-I/ARB, Beta Blocker, Aldosterone Antagonist, Loop Diuretics, Nitrates, CCB, Sodium, GFR (MDRD), Glucose, and HGB.
Stepwise selection: levels for entry into and staying in the model are both 0.1.

Table S34. Multivariate Cox Proportional Hazards Regression on incident HF, MI and all-cause Death, with age, sex, dichotomized Singulex hsTnl and other covariates*
Patients with obstructive CAD only (N=619)

Parameter	Parameter Estimate	Standard Error	P-Value	Hazard Ratio	95% CI
Age, years	0.04416	0.00737	<0.001	1.05	[1.03, 1.06]
Male sex	0.18250	0.17895	0.31	1.20	[0.85, 1.70]
HsTnl ≥99th percentile (ng/L)	0.53407	0.15903	<0.001	1.71	[1.25, 2.33]
Heart Rate (Beat/min)	0.01133	0.00526	0.03	1.01	[1.00, 1.02]
Atrial Fibrillation/Flutter	0.28680	0.16504	0.08	1.33	[0.96, 1.84]
Coronary Artery Disease	0.51709	0.17106	0.003	1.68	[1.20, 2.35]
COPD	0.62660	0.16930	<0.001	1.87	[1.34, 2.61]
Diabetes mellitus	0.56140	0.14741	<0.001	1.75	[1.31, 2.34]
ACE-I/ARB	-0.25920	0.15289	0.09	0.77	[0.57, 1.04]
Loop diuretics	0.69017	0.17117	<0.001	1.99	[1.43, 2.79]
NT-ProBNP (pg/mL)	0.0000422	7.92824E-6	<0.001	1.00	[1.00, 1.00]

ACE-I= Angiotensin converting enzyme inhibitors, CI= confidence interval, COPD= chronic obstructive pulmonary disease, HF= heart failure, Hgb=hemoglobin, hsTn= high sensitivity troponin, MI= myocardial infarction, NT-proBNP= amino-terminal pro-B type natriuretic peptide.

***Other Covariates include**: Heart Rate, Systolic Blood Pressure, Diastolic Blood Pressure, Atrial Fibrillation/Flutter, Hypertension, Coronary Artery Disease, Heart Failure, COPD, Diabetes type I/type II, CKD, ACE-I/ARB, Beta Blocker, Aldosterone Antagonist, Loop Diuretics, Nitrates, CCB, Sodium, GFR (MDRD), Glucose, and HGB.
Stepwise selection: levels for entry into and staying in the model are both 0.1.

Table S35. Multivariate Cox Proportional Hazards Regression on composite outcome of MI and all-cause death, with age, sex, dichotomized Singulex hsTnl and other covariates*
Patients with obstructive CAD only (N=619)

Parameter	Parameter Estimate	Standard Error	P-Value	Hazard Ratio	95% CI
Age, years	0.04956	0.00890	<0.001	1.05	[1.03, 1.07]
Male sex	-0.06860	0.20345	0.74	0.93	[0.63, 1.39]
HsTnl ≥99th percentile (ng/L)	0.72200	0.18925	<0.001	2.06	[1.42, 2.98]
Heart Rate (Beat/min)	0.01150	0.00629	0.07	1.01	[1.00, 1.02]
Systolic Blood Pressure (mmHg)	-0.01281	0.00401	0.001	0.99	[0.98, 1.00]
Coronary Artery Disease	0.37047	0.20385	0.07	1.45	[0.97, 2.16]
COPD	0.74653	0.19221	<0.001	2.11	[1.45, 3.07]
Nitrates	0.49807	0.18320	0.01	1.65	[1.15, 2.36]
MPO (pmol/L)	0.0002693	0.0001173	0.02	1.00	[1.00, 1.00]
NT-ProBNP (pg/mL)	0.0000230	0.0000127	0.07	1.00	[1.00, 1.00]
Cystatin C (mg/L)	0.26008	0.09794	0.01	1.30	[1.07, 1.57]

CI= confidence interval, COPD= chronic obstructive pulmonary disease, hsTn= high sensitivity troponin, MI= myocardial infarction, MPO=myeloperoxidase, NT-proBNP= amino-terminal pro-B type natriuretic peptide.

***Other Covariates include**: Heart Rate, Systolic Blood Pressure, Diastolic Blood Pressure, Atrial Fibrillation/Flutter, Hypertension, Coronary Artery Disease, Heart Failure, COPD, Diabetes type I/type II, CKD, ACE-I/ARB, Beta Blocker, Aldosterone Antagonist, Loop Diuretics, Nitrates, CCB, Sodium, GFR (MDRD), Glucose, and HGB.
Stepwise selection: levels for entry into and staying in the model are both 0.1

Table S36. Multivariate Cox Proportional Hazards Regression on Composite outcome of MI and CV death, with age, sex, dichotomized Singulex hsTnl and other covariates*
Patients with obstructive CAD only (N=619)

Parameter	Parameter Estimate	Standard Error	P-Value	Hazard Ratio	95% CI
Age, years	0.04610	0.00935	<0.001	1.05	[1.03, 1.07]
Male sex	0.02251	0.21882	0.92	1.02	[0.67, 1.57]
HsTnl ≥99th percentile (ng/L)	0.72485	0.19928	<0.001	2.06	[1.40, 3.05]
Heart Rate (Beat/min)	0.01348	0.00664	0.04	1.01	[1.00, 1.03]
Systolic Blood Pressure (mmHg)	-0.01254	0.00420	0.003	0.99	[0.98, 1.00]
Coronary Artery Disease	0.36030	0.21305	0.09	1.43	[0.94, 2.18]
COPD	0.64983	0.20518	0.002	1.92	[1.28, 2.86]
Nitrates	0.53763	0.19102	0.01	1.71	[1.18, 2.49]
Hemoglobin, g/L	-0.10180	0.06062	0.09	0.90	[0.80, 1.02]
MPO (pmol/L)	0.0002180	0.0001255	0.08	1.00	[1.00, 1.00]
NT-ProBNP (pg/mL)	0.0000232	0.0000126	0.07	1.00	[1.00, 1.00]
Cystatin C (mg/L)	0.22073	0.10484	0.04	1.25	[1.02, 1.53]

CI= confidence interval, COPD= chronic obstructive pulmonary disease, CV= cardiovascular, hsTn= high sensitivity troponin, Hgb=hemoglobin, MI= myocardial infarction, MPO=myeloperoxidase, NT-proBNP= amino-terminal pro-B type natriuretic peptide.

***Other Covariates include**: Heart Rate, Systolic Blood Pressure, Diastolic Blood Pressure, Atrial Fibrillation/Flutter, Hypertension, Coronary Artery Disease, Heart Failure, COPD, Diabetes type I/type II, CKD, ACE-I/ARB, Beta Blocker, Aldosterone Antagonist, Loop Diuretics, Nitrates, CCB, Sodium, GFR (MDRD), Glucose, and HGB. Stepwise selection: levels for entry into and staying in the model are both 0.1.

Table S37. Multivariate Cox Proportional Hazards Regression on incident MI, with age, sex, dichotomized Singulex hsTnl and other covariates*
Patients with non-obstructive CAD only (N=226)

Parameter	Parameter Estimate	Standard Error	P-Value	Hazard Ratio	95% CI
Age, years	-0.05303	0.02442	0.03	0.95	[0.90, 0.99]
Male sex	0.94914	0.55781	0.09	2.58	[0.87, 7.71]
HsTnl ≥99th percentile (ng/L)	2.12898	0.56666	<0.001	8.41	[2.77, 25.52]
Diabetes mellitus	1.18248	0.51107	0.02	3.26	[1.20, 8.88]
CCB	1.23025	0.53775	0.02	3.42	[1.19, 9.82]
Hemoglobin, g/L	-0.42977	0.16196	0.01	0.65	[0.47, 0.89]
MPO (pmol/L)	0.0008414	0.0002317	<0.001	1.00	[1.00, 1.00]

CI= confidence interval, CCB= calcium channel blocker, hsTn= high sensitivity troponin, Hgb=hemoglobin, MI= myocardial infarction, MPO=myeloperoxidase.

***Other Covariates include**: Heart Rate, Systolic Blood Pressure, Diastolic Blood Pressure, Atrial Fibrillation/Flutter, Hypertension, Coronary Artery Disease, Heart Failure, COPD, Diabetes type I/type II, CKD, ACE-I/ARB, Beta Blocker, Aldosterone Antagonist, Loop Diuretics, Nitrates, CCB, Sodium, GFR (MDRD), Glucose, and HGB.
Stepwise selection: levels for entry into and staying in the model are both 0.1.

Table S38. Cox Proportional Hazard Regression on incident CHF with age, sex, dichotomized singulex high-sensitivity troponin and other covariates*
Patients with Non-obstructive CAD (N = 226)

Parameter	Parameter Estimate	Standard Error	P-Value	Hazard Ratio	95% CI
Age, years	0.01032	0.01514	0.50	1.01	[0.98, 1.04]
Male sex	0.70930	0.34501	0.04	2.03	[1.03, 4.00]
HsTnl ≥99th percentile (ng/L)	0.47886	0.32652	0.14	1.61	[0.85, 3.06]
Heart Failure	1.15586	0.30707	<0.001	3.18	[1.74, 5.80]
NT-ProBNP (pg/mL)	0.0000705	0.0000242	0.004	1.00	[1.00, 1.00]
Cystatin C (mg/L)	0.41525	0.14238	0.004	1.51	[1.15, 2.00]

CI= confidence interval, HF= heart failure, hsTn= high sensitivity troponin, NT-proBNP= amino-terminal pro-B type natriuretic peptide.

***Other Covariates include**: Heart Rate, Systolic Blood Pressure, Diastolic Blood Pressure, Atrial Fibrillation/Flutter, Hypertension, Coronary Artery Disease, Heart Failure, COPD, Diabetes type I/type II, CKD, ACE-I/ARB, Beta Blocker, Aldosterone Antagonist, Loop Diuretics, Nitrates, CCB, Sodium, GFR (MDRD), Glucose, and HGB.
Stepwise selection: levels for entry into and staying in the model are both 0.1.

Table S39. Multivariate Cox Proportional Hazards Regression on CV Mortality, with age, sex, dichotomized Singulex hsTnl and other covariates*
Patients with non-obstructive CAD only (N=226)

Parameter	Parameter Estimate	Standard Error	P-Value	Hazard Ratio	95% CI
Age, years	0.03557	0.03166	0.26	1.04	[0.97, 1.10]
Male sex	2.22663	0.71965	0.002	9.27	[2.26, 37.98]
HsTnl ≥99th percentile (ng/L)	1.28013	0.58344	0.03	3.60	[1.15, 11.29]
Diabetes mellitus	1.17336	0.54995	0.03	3.23	[1.10, 9.50]
Loop diuretics	1.70533	0.64350	0.01	5.50	[1.56, 19.43]
Hemoglobin, g/L	-0.81344	0.25565	0.001	0.44	[0.27, 0.73]
Cystatin C (mg/L)	0.43953	0.23676	0.06	1.55	[0.98, 2.47]

CI= confidence interval, CV=cardiovascular, hsTn= high sensitivity troponin, Hgb=hemoglobin.

***Other Covariates include**: Heart Rate, Systolic Blood Pressure, Diastolic Blood Pressure, Atrial Fibrillation/Flutter, Hypertension, Coronary Artery Disease, Heart Failure, COPD, Diabetes type I/type II, CKD, ACE-I/ARB, Beta Blocker, Aldosterone Antagonist, Loop Diuretics, Nitrates, CCB, Sodium, GFR (MDRD), Glucose, and HGB.
Stepwise selection: levels for entry into and staying in the model are both 0.1.

Table S40. Multivariate Cox Proportional Hazards Regression on All-Cause Mortality, with age, sex, dichotomized Singulex hsTnl and other covariates*
Patients with non-obstructive CAD only (N=226)

Parameter	Parameter Estimate	Standard Error	P-Value	Hazard Ratio	95% CI
Age, years	0.03730	0.02932	0.20	1.04	[0.98, 1.10]
Male sex	2.14214	0.63573	<0.001	8.52	[2.45, 29.61]
HsTnl ≥99th percentile (ng/L)	0.81073	0.51093	0.11	2.25	[0.83, 6.12]
Diabetes mellitus	0.83851	0.50902	0.10	2.31	[0.85, 6.27]
Loop diuretics	1.33269	0.53008	0.01	3.79	[1.34, 10.71]
Hemoglobin, g/L	-0.76717	0.21458	<0.001	0.46	[0.30, 0.71]
MPO (pmol/L)	0.0004192	0.0002104	0.05	1.00	[1.00, 1.00]

CI= confidence interval, hsTn= high sensitivity troponin, Hgb=hemoglobin, MPO=myeloperoxidase.

Other Covariates include: Heart Rate, Systolic Blood Pressure, Diastolic Blood Pressure, Atrial Fibrillation/Flutter, Hypertension, Coronary Artery Disease, Heart Failure, COPD, Diabetes type I/type II, CKD, ACE-I/ARB, Beta Blocker, Aldosterone Antagonist, Loop Diuretics, Nitrates, CCB, Sodium, GFR (MDRD), Glucose, and HGB.
Stepwise selection: levels for entry into and staying in the model are both 0.1.

Table S41. Multivariate Cox Proportional Hazards Regression on incident HF, MI and Death, with age, sex, dichotomized Singulex hsTnl and other covariates*
Patients with non-obstr1uctive CAD only (N=226)

Parameter	Parameter Estimate	Standard Error	P-Value	Hazard Ratio	95% CI
Age, years	-0.00500	0.01460	0.73	1.00	[0.97, 1.02]
Male sex	0.76690	0.34378	0.03	2.15	[1.10, 4.22]
HsTnl ≥99th percentile (ng/L)	0.46906	0.30468	0.12	1.60	[0.88, 2.90]
Heart Rate (Beat/min)	0.02041	0.00950	0.03	1.02	[1.00, 1.04]
Heart Failure	0.57757	0.30341	0.06	1.78	[0.98, 3.23]
COPD	0.84378	0.32962	0.01	2.33	[1.22, 4.44]
Diabetes mellitus	0.89801	0.31310	0.004	2.45	[1.33, 4.53]
Hemoglobin, g/L	-0.19368	0.08953	0.03	0.82	[0.69, 0.98]
MPO (pmol/L)	0.0004121	0.0001590	0.01	1.00	[1.00, 1.00]
NT-ProBNP (pg/mL)	0.0000755	0.0000224	<0.001	1.00	[1.00, 1.00]

CI= confidence interval, COPD= chronic obstructive pulmonary disease, CV= cardiovascular, hsTn= high sensitivity troponin, HF= heart failure, Hgb=hemoglobin, MI= myocardial infarction, MPO=myeloperoxidase, NT-proBNP= amino-terminal pro-B type natriuretic peptide.

Other Covariates include: Heart Rate, Systolic Blood Pressure, Diastolic Blood Pressure, Atrial Fibrillation/Flutter, Hypertension, Coronary Artery Disease, Heart Failure, COPD, Diabetes type I/type II, CKD, ACE-I/ARB, Beta Blocker, Aldosterone Antagonist, Loop Diuretics, Nitrates, CCB, Sodium, GFR (MDRD), Glucose, and HGB.
Stepwise selection: levels for entry into and staying in the model are both 0.1.

Table S42. Multivariate Cox Proportional Hazards Regression on composite outcome of MI and all-cause death, with age, sex, dichotomized Singulex hsTnl and other covariates*
Patients with non-obstructive CAD only (N=226)

Parameter	Parameter Estimate	Standard Error	P-Value	Hazard Ratio	95% CI
Age, years	-0.01432	0.01845	0.44	0.99	[0.95, 1.02]
Male sex	0.96222	0.41838	0.02	2.62	[1.15, 5.94]
HsTnl ≥99th percentile (ng/L)	1.28726	0.36236	<0.001	3.62	[1.78, 7.37]
Heart Rate (Beat/min)	0.02238	0.01089	0.04	1.02	[1.00, 1.04]
Diabetes mellitus	0.73221	0.39189	0.06	2.08	[0.96, 4.48]
Beta blocker	0.82864	0.46786	0.08	2.29	[0.92, 5.73]
Hemoglobin, g/L	-0.53620	0.12859	<0.001	0.58	[0.45, 0.75]
MPO (pmol/L)	0.0004676	0.0001863	0.01	1.00	[1.00, 1.00]

CI= confidence interval, hsTn= high sensitivity troponin, Hgb=hemoglobin, MI= myocardial infarction, MPO=myeloperoxidase.

***Other Covariates include**: Heart Rate, Systolic Blood Pressure, Diastolic Blood Pressure, Atrial Fibrillation/Flutter, Hypertension, Coronary Artery Disease, Heart Failure, COPD, Diabetes type I/type II, CKD, ACE-I/ARB, Beta Blocker, Aldosterone Antagonist, Loop Diuretics, Nitrates, CCB, Sodium, GFR (MDRD), Glucose, and HGB.
Stepwise selection: levels for entry into and staying in the model are both 0.1

Table S43. Multivariate Cox Proportional Hazards Regression on Composite outcome of MI and CV death, with age, sex, dichotomized Singulex hsTnl and other covariates
Patients with non-obstructive CAD only (N=226)

Parameter	Parameter Estimate	Standard Error	P-Value	Hazard Ratio	95% CI
Age, years	-0.01671	0.01835	0.36	0.98	[0.95, 1.02]
Male sex	1.02409	0.42695	0.02	2.78	[1.21, 6.43]
HsTnl ≥99th percentile (ng/L)	1.54770	0.39417	<0.001	4.70	[2.17, 10.18]
Heart Rate (Beat/min)	0.02230	0.01144	0.05	1.02	[1.00, 1.05]
Diabetes mellitus	1.18935	0.37855	0.002	3.28	[1.56, 6.90]
Hemoglobin, g/L	-0.53097	0.13931	<0.001	0.59	[0.45, 0.77]
MPO (pmol/L)	0.0005873	0.0001950	0.003	1.00	[1.00, 1.00]

CI= confidence interval, CV=cardiovascular, hsTn= high sensitivity troponin, Hgb=hemoglobin, MI= myocardial infarction, MPO=myeloperoxidase,

Other Covariates include: Heart Rate, Systolic Blood Pressure, Diastolic Blood Pressure, Atrial Fibrillation/Flutter, Hypertension, Coronary Artery Disease, Heart Failure, COPD, Diabetes type I/type II, CKD, ACE-I/ARB, Beta Blocker, Aldosterone Antagonist, Loop Diuretics, Nitrates, CCB, Sodium, GFR (MDRD), Glucose, and HGB. Stepwise selection: levels for entry into and staying in the model are both 0.1.

Table S44. Proportionality Test Result for MI, HF, CV, Death, MI/CV death, MI/all cause death, HF/MI/all-cause death. (Full Cohort N = 991)

Model	Wald Chi-Square	P-Value
MI	14.2692	0.16
HF	10.0601	0.35
CV	15.3450	0.12
Death	19.7582	0.03
MI/CV death	15.2753	0.17
MI/all cause death	16.8244	0.16
HF/MI/all cause death	13.9015	0.18

CV= cardiovascular, HF= heart failure, MI= myocardial infarction, N= number of patients

Figure S1. Kaplan-Meier Curve for Cumulative Incidence of MI With Obstructive CAD (≥70% stenosis).

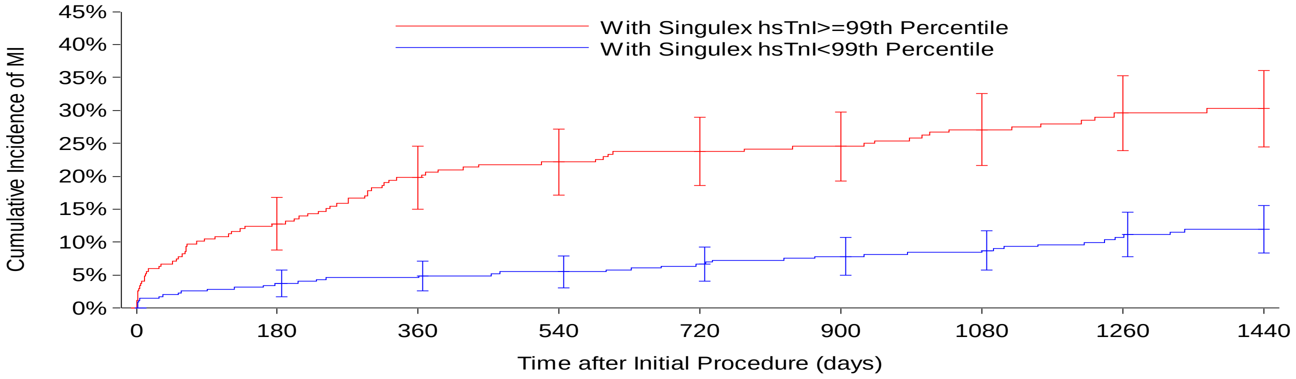

Time since coronary angiography	0	180	360	540	720	900	1080	1260	1440
hsTnI≥99th Percentile	269	228	204	197	189	181	170	116	100
% Cumulative Incidence	0.00%	12.77%	19.79%	22.15%	23.74%	24.56%	27.08%	29.61%	30.26%
hsTnI<99th Percentile	350	334	329	323	316	312	306	224	201
% Cumulative Incidence	0.00%	3.73%	4.89%	5.47%	6.65%	7.83%	8.72%	11.14%	11.95%

Figure S2. Kaplan-Meier Curve for Cumulative Incidence of MI Obstructive (≥70% stenosis) vs. non-obstructive CAD (<70 but > 0% stenosis)

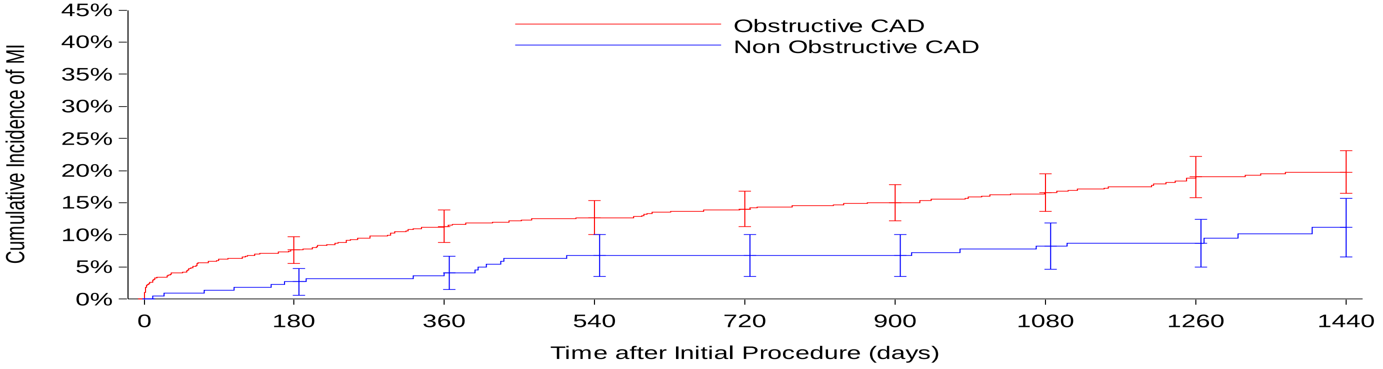

Time since coronary angiography	0	180	360	540	720	900	1080	1260	1440
obstructive CAD	619	562	533	520	505	493	476	340	301
% Cumulative Incidence	0.00%	7.64%	11.30%	12.63%	13.98%	15.01%	16.57%	19.03%	19.77%
non-obstructive CAD	226	217	212	203	200	195	191	124	98
% Cumulative Incidence	0.00%	2.68%	4.03%	6.77%	6.77%	6.77%	8.21%	8.69%	11.11%

Figure S3. Kaplan-Meier Curve for Cumulative Incidence of MI With non-obstructive CAD (<70% but >0% stenosis)

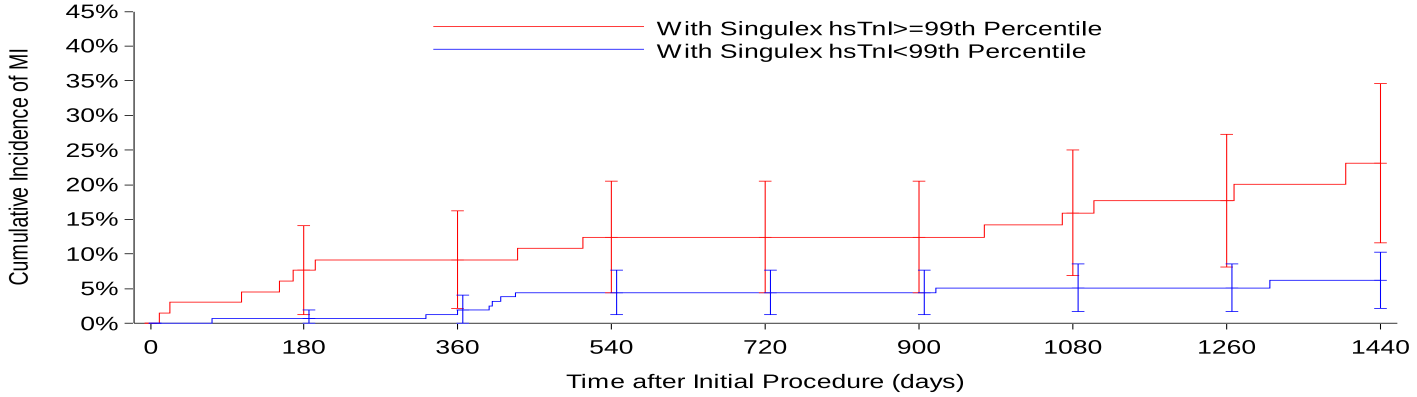

Time since coronary angiography	0	180	360	540	720	900	1080	1260	1440
hsTnI>=99th Percentile	66	60	57	54	52	51	48	34	26
% Cumulative Incidence	0.00%	7.65%	9.19%	12.43%	12.43%	12.43%	15.93%	17.68%	23.06%
hsTnI<99th Percentile	160	157	155	149	148	144	143	90	72
% Cumulative Incidence	0.00%	0.63%	1.90%	4.45%	4.45%	4.45%	5.11%	5.11%	6.21%
